# Genome-Wide Identification and Function Analyses of Heat Shock Transcription Factors in Potato

**DOI:** 10.3389/fpls.2016.00490

**Published:** 2016-04-19

**Authors:** Ruimin Tang, Wenjiao Zhu, Xiaoyan Song, Xingzhong Lin, Jinghui Cai, Man Wang, Qing Yang

**Affiliations:** Biochemistry and Molecular Biology, College of Life Sciences, Nanjing Agricultural UniversityNanjing, China

**Keywords:** heat shock transcription factors, potato, bioinformatics, abiotic stresses, gene expression, co-expression network

## Abstract

Heat shock transcription factors (Hsfs) play vital roles in the regulation of tolerance to various stresses in living organisms. To dissect the mechanisms of the Hsfs in potato adaptation to abiotic stresses, genome and transcriptome analyses of *Hsf* gene family were investigated in *Solanum tuberosum* L. Twenty-seven StHsf members were identified by bioinformatics and phylogenetic analyses and were classified into A, B, and C groups according to their structural and phylogenetic features. StHsfs in the same class shared similar gene structures and conserved motifs. The chromosomal location analysis showed that 27 *Hsfs* were located in 10 of 12 chromosomes (except chromosome 1 and chromosome 5) and that 18 of these genes formed 9 paralogous pairs. Expression profiles of *StHsfs* in 12 different organs and tissues uncovered distinct spatial expression patterns of these genes and their potential roles in the process of growth and development. Promoter and quantitative real-time polymerase chain reaction (qRT-PCR) detections of *StHsfs* were conducted and demonstrated that these genes were all responsive to various stresses. *StHsf004, StHsf007, StHsf009, StHsf014*, and *StHsf019* were constitutively expressed under non-stress conditions, and some specific *Hsfs* became the predominant *Hsfs* in response to different abiotic stresses, indicating their important and diverse regulatory roles in adverse conditions. A co-expression network between *StHsfs* and *StHsf* -co-expressed genes was generated based on the publicly-available potato transcriptomic databases and identified key candidate *StHsfs* for further functional studies.

## Introduction

Plants always suffer from various adverse environmental stresses in their growth and development periods and have developed special mechanisms to respond to the changing adverse conditions (Wang et al., 2014). These response mechanisms are regulated by substantial regulatory interactions and coordination of distinct signal transduction pathways in plant responses to intricate abiotic stresses (Singh et al., 2002; Katagiri, 2004; Ahuja et al., 2010; Mittler et al., 2012).

Numerous signaling components and downstream effectors participate in different biological reactions and connect the reaction pathways into a regulatory network. Among these components, various transcription factors play central roles in regulation of the stress-induced responses (Kotak et al., 2007a). Heat shock transcription factors (Hsfs), one sort of the most important transcription regulators, are the terminal elements of the signal transduction chain and mediate the activation of genes responsive to various abiotic stresses including heat stress, drought stress and a large number of chemical stressors (such as Cd^2+^ and salicylate) (Baniwal et al., 2004). Hsfs can regulate the transcription of *Hsp* genes by binding specifically with heat shock elements (HSE) in *Hsp* gene promoters, and Hsps subsequently protect cells against stress impairment and participate in protein folding (Morimoto, 1993; Schöffl et al., 1998; Hartl and Hayer-Hartl, 2002). Several results have already confirmed that Hsfs are responsible to other abiotic stresses apart from heat stress. For instance, *HsfA1e, HsfA3, HsfA4a, HsfB2a*, and *HsfC1* are strongly induced by cold, salt and osmotic stresses in *Arabidopsis*, suggesting that Hsfs are significant regulatory molecules in the complex network of response pathways (Miller and Mittler, 2006; Kilian et al., 2007; Swindell et al., 2007; Zhang et al., 2015).

Analogous to other transcription factors, a typical feature at the N-terminus of Hsfs with a helix-turn-helix structure is their DNA-binding domain (DBD), which is the best preserved motif and can be used to accurately recognize the core repeating units of HSE (5′-nGAAn-3′) (Damberger et al., 1994; Döring et al., 2000; Ma et al., 2014). Another conserved domain adjacent to DBD motif is the oligomerization domain (OD). This domain consists of hydrophobic heptad repeats (HR-A and HR-B) which can form a curly structure to activate the formation of an Hsf trimer to combine with *Hsp* genes' promoter efficiently (Sorger and Nelson, 1989; Wu, 1995; Lyck et al., 1997; Scharf et al., 2012). Besides DBD and OD, a nuclear localization signal (NLS) characterized by a cluster of arginine and lysine residues and a nuclear export signal (NES) rich in leucine residues are two structures close to the C-terminal of Hsfs (Lyck et al., 1997; Scharf et al., 2012). In most class A Hsfs, there is a C-terminal activation domain (CTAD) with amino acids activation sequence (AHA), in which the first “A” represents aromatic amino acids W, F, Y, “H” represents large hydrophobic amino acids L, I, V, M, and the second “A” stands for acidic amino acids D and E (Xue et al., 2014). Instead, nearly all class B members in the C-terminal region contain a peptide with four amino acids: -LFGV-, which is considered as the core of a repression sequence and widely exists in other transcription factors, like *ABI3/VP1* and *MYB/GRAS* (Ikeda and Ohme-Takagi, 2009), but how this peptide interacts with other domains and how to play a repression role in the process of transcription remain obscure (Czarnecka-Verner et al., 2004).

Since the development of the sequencing technology, Hsf members from various species have been identified, such as yeast (Wiederrecht et al., 1988), *Arabidopsis* (Nover et al., 2001), tomato (Mishra et al., 2002), rice (Wang et al., 2009), and pepper (Guo et al., 2015), which contains 1, 21, 24, 25, and 25 members, respectively. So far, only four kinds of Hsfs were discovered in animals, including Hsf1, Hsf2, Hsf3, and Hsf4 (Zheng et al., 2008; Akerfelt et al., 2010). Compared with the animal Hsfs, plant Hsfs are more diverse, partially redundant, and functionally flexible (Miller and Mittler, 2006). The components of Hsf members have noticeable difference among different plant species. Although Hsfs in Dicotyledonous and monocotyledonous have high similarity, some types of Hsfs express specifically: *HsfA9* only exists in dicotyledonous plants and *HsfC2* only exists in monocotyledonous plants (Xue et al., 2014). Even though the composition of Hsfs in different plants is clear, the functions of these Hsfs have not been determined completely.

Many researches have demonstrated that the function of *Hsf* members differs from the species in plant development and in the interlaced stress response pathways (Xue et al., 2014). HsfA3 has been found as an important player in the crosstalk of heat and drought stresses in *Arabidopsis* while similar function of HsfA3 was not detected in tomato (Von Koskull-Döring et al., 2007). HsfA9 was considered to play a specific role in seed development in many plant species like *Arabidopsis*, tomato and tobacco whereas HsfA7 appears to perform the parallel function as HsfA9 in rice (Kotak et al., 2007b; Von Koskull-Döring et al., 2007; Chauhan et al., 2011). Although HsfA9 was well known in regulating the seed maturation, it has also been described to involve in the development of pericarp and placenta in pepper (Guo et al., 2015).

Multiple signal transduction pathways have been demonstrated to regulate the *Hsp* expression by activating Hsfs to bind to the heat shock element of heat shock genes, but the molecular pathway is still elusive for the entire function of Hsfs and their related genes in different stresses (Singh et al., 2002; Zhang et al., 2015). Recently, the package of WGCNA (Weighted correlation network analysis) has provided a possible method to analyze the response of Hsfs and the correlated genes to different abiotic stresses using numerous microarray datasets or transcription data (Langfelder and Horvath, 2008). WGCNA package, which was designed for clustering the related genes into a module based on the tissue types and correlated biological pathways, has been used in mouse, yeast and many kinds of plant species (Langfelder and Horvath, 2008). By this way, Downs et al. (2013) identified several tissue-specific modules and signal pathway-specific modules in maize, and also detected the potential molecular components in these modules. Zhang et al. (2015) created a co-expression network using this package to investigate the relationship between Hsfs and Hsps in Populus and discover the possible regulatory mechanisms among them.

Potato is the fourth most important food crop following wheat, corn and rice in the world. During the potato field cultivation, adverse stresses often do great harm to their growth and lead to a decline in potato output. Therefore, study on stress resistance and exploitation of related genes is becoming more and more important and urgent for potato breeding and production. To our knowledge, there are no reports for identification and functional elucidation of potato Hsfs to date. In this study, a bioinformatics analysis was used to conduct the genome-wide identification of potato Hsf family members with the open access databases. In order to shed light into their underlying roles, the expression profiles of these deduced Hsf members were performed in various tissues and also in response to heat and other abiotic stresses (drought and cold stress). Our analysis indicated that some *Hsf* genes exhibited specific expression patterns in response to distinct stresses. A co-expression network between *StHsfs* and their correlated genes helped to identify molecular components in the same pathways and select the candidate genes for further research.

## Materials and methods

### Plant materials and growth conditions

The potato plantlets (*Solanum tuberosum* L.) from our own laboratory were cultured in MS medium (Murashige and Skoog, 1962) containing 3% sucrose and 0.8% agar at pH 5.8, and maintained under 16 h light/8 h dark regime at 22 ± 1°C. Subculture was conducted every 4 weeks. The 1-month-old plantlets were then transferred into tubes with half strength Hoagland solution with aeration in illumination incubator under 16 h light/8 h dark regime at 22 ± 1°C for another 2 weeks before being treated with heat, drought and cold stresses. For temperature stress treatments, the plantlets were exposed to 4 or 35°C; for drought treatments, the plantlets were incubated with 3% PEG-6000. Under these different stress treatments, the 2nd fully expanded leaves from the top of plantlets leaves were collected at 0, 2, 6, and 24 h. All harvested samples were immediately immersed in liquid nitrogen and stored at −80°C prior to RNA extraction.

### Identification of the Hsf members in potato

The sequences of potato *Hsf* members were firstly searched in the Plant Transcription Factor Database (Riaño-Pachón et al., 2007). To identify a complete list of potato *Hsf* genes, the Hidden Markov Model (HMM) profile and consensus pattern of the Hsf DBD (PF00447) were downloaded from the Pfam database (http://pfam.xfam.org/search) to obtain the conserved domain. Protein sequences of Hsfs conserved domain and potato genome protein sequences were aligned by BLASTp research in the NCBI protein database (http://blast.ncbi.nlm.nih.gov/blast.cgi) and Spud DB Potato Genomics Resources (http://solanaceae.plantbiology.msu.edu/) with *E*-value of 0.001 to screen candidate Hsfs with homologous amino acids sequences preliminarily. These candidate genes were analyzed using the domain identification function of the Pfam database (*E* = 1.0) to remove the Hsfs without the conserved domain sequences. The multiple protein sequence alignment of the candidate genes was then performed by Clustal W which provided by MEGA 4.0 to remove repetitive sequences. Length of amino acids sequences, theoretical molecular weights, isoelectric points and grand average of hydropathicity of deduced Hsf proteins were calculated using ProtParam tools provided by the ExPasy website (http://web.expasy.org/tools/protparam). The DBD and heptad repeat region (HR-A/B) sequences of Hsf proteins were aligned by DNAMAN software, respectively. The information of chromosome location and genomic length of the predicted genes was obtained from the Spud DB Potato Genomics Resources.

### Phylogenetic analysis and classification of these identified Hsfs

The heptad repeat region alignment was used to preliminarily classify these identified Hsfs into three classes. The application of phylogenetic analysis helped to further divide potato Hsf members into different groups based on the Hsf classification scheme of other dicotyledon plants like tomato, cucumber, soybean and *Arabidopsis* (Scharf et al., 2012; Xue et al., 2014). In total, 145 Hsf protein sequences from potato and other four species (Nover et al., 2001; Baniwal et al., 2004; Chung et al., 2013; Zhou et al., 2013) were used for construction of a phylogenetic tree by Clustal W alignment and the unrooted Neighbor-Joining method with 1000 bootstrap replicates using MEGA 4.0.

### Chromosomal location of *StHsfs*

All identified *StHsf* genes were mapped to potato chromosomes using MapInspect software (http://www.plantbreeding.wur.nl/uk/software-mapinspect.html) based on the information available at the website of Spud DB Potato Genomics Resources (http://solanaceae.plantbiology.msu.edu/). Tandem duplications of paralogous *Hsf* genes and *Hsf* gene clusters in the potato genome were marked according to the method used by Yuan et al. (2015) and Zhang et al. (2015), respectively.

### Analyses of gene structures, conserved motifs, and response elements

The exon and intron organization of potato *Hsfs* was depicted by comparing the coding sequences of *StHsfs* with their corresponding genomic sequences using Gene Structure Display Server online (http://gsds.cbi.pku.edu.cn) (Guo et al., 2007). The program of Multiple Em for Motif Elicitation online (http://meme.sdsc.edu/meme/intro.html) (Bailey et al., 2009) was utilized to analyze the conserved motifs of the predicted StHsf proteins with the following parameters: the number of repetitions, any; the maximum number of motifs, 20; and the optimum width of each motif, between 6 and 300 residues. The analysis of response elements in *StHsf* genes promoter region (up to −2000 bp upstream of the coding sequences), which are available in the potato genome database of Phytozome v10.3 (http://www.phytozome.net), was conducted by the PLACE website (http://www.dna.affrc.go.jp/PLACE/) (Higo et al., 1999).

### RNAseq analysis and co-expression network construction

The expression pattern analysis of *StHsf* genes in different tissues was carried out using the potato RNAseq data (PGSC, 2011) downloaded from Spud DB Potato Genomics Resources (http://solanaceae.plantbiology.msu.edu/). The normalized expression data from the database were calculated as log_2_ fold change and displayed in a heatmap using the R Project software.

A co-expression network between *StHsfs* and their correlated genes was constructed by weighted correlation network analysis (WGCNA), which provides a systematic method to investigate the potential related genes in the same pathway using the microarray data or RNAseq data (Langfelder and Horvath, 2008). The genes whose maximum expression values in the RNAseq data less than 5 were filtered out. Then the expression data of the remained genes was used to calculate the adjacency matrix between genes in all samples according to the Pearson correlation coefficient (Langfelder and Horvath, 2008). The adjacent values between the two genes can be expressed in the following formula (Langfelder and Horvath, 2008):

$${\text{a}}_{ij}={\left|\frac{\left(1+s_{\;ij}\right)}{2}\right|}^{\beta }$$

In the formula, a_*ij*_ represents the adjacency value between gene *i* and gene *j*; s_*ij*_ is the Pearson correlation coefficient between gene *i* and *j*; β represents the weight value. WGCNA method was utilized to convert the adjacency value into the Topological overlap value (TO) which can represent the correlation of the genes in the network. The dissimilarity matrix, the inverse matrix of TO value, was hierarchical clustered to represent the genetic link network. The dynamic treecut algorithm was used to cut the hierarchical clustering tree and the obtained branches represent different modules (Langfelder et al., 2008). The network was graphically presented using Cytoscape software with the TOM (Topological overlap matrix) value 0.07 (Shannon et al., 2003).

### RNA isolation and expression analysis of selected *StHsfs* using quantitative real-time PCR

Total RNA from stress-treated leaves was isolated using the RNA Extract kit (TaKaRa, Dalian, China) with the treatment of RNase-free DNase I to erase the genomic DNA (TaKaRa, Dalian, China). After the examination of the RNA integrity and concentration, 0.5 μg RNA per sample was used to synthesize the first-strand cDNA using the Transcriptor First Strand cDNA Synthesis Kit (Roche, Mannheim, Germany). One micro liter of the synthetic cDNA was diluted by 9 μL nuclease-free water before the qRT-PCR analysis.

Quantitative real-time polymerase chain reaction (qRT-PCR) was carried out with the FastStart Universal SYBR Green Master (ROX) (Roche, Mannheim, Germany) on an ABI 7500 Real Time PCR System (Applied Biosystems, Foster City, CA, USA). The specificity of these primers which designed by Beacon designer software was tested by blast tool in NCBI and dissociation curve analysis. Each PCR reaction was conducted in a 20 μl reaction volume containing 10 μL SYBR-Green, 6.8 μL ddH_2_O, 2 μL diluted template and 0.6 μL 10 μM solution of each primer. The PCR cycling program consisted of 50°C for 2 min, 95°C for 10 min followed by 40 cycles at 95°C for 5 s, and 60°C for 35 s. The relative expression of each selected gene was normalized using the method of 2^−ΔΔCt^ against the reference gene *EF1*α (Yang et al., 2013), whose transcript level keeps relatively steady under different conditions. For each sample, two experimental replicates and three biological repeats were performed to make sure the results reliable. Results were presented as means ± SD. The method of Dunnett's two-tailed *t*-test was used to conduct the statistical analyses of RT-PCR results, and the statistical significant differences were shown at *p* ≤ 0.05 (marked ^*^) and *p* ≤ 0.01 (marked ^**^).

## Results

### Twenty-seven potato Hsf members were identified and classified into three classes

Potato Hsf members were searched from the Plant Transcription Factor Database, NCBI GenBank database and Spud DB Potato Genomics Resources. Twenty-seven full-length *Hsf* genes were identified as potential members of potato Hsf family after removing the redundant and non-full length sequences according to the consensus pattern of the Hsf DBD in the Pfam database (Supplementary S1). The results of multisequencing alignments of the DBD and heptad repeat regions (HR-A/B) of potato Hsfs presented high conservation in the DBD and the HR-A/B regions of these Hsf members (Supplementary Figures S1, S2).

Based on the numbers of amino acids inserted in HR-A core and HR-B core of StHsfs, StHsfs were classified into A, B, and C three major classes (Supplementary Figure S2). Class A is comprised of 18 proteins, each containing 21 amino acids between HR-A and HR-B except StHsf005 and StHsf006 which respectively have 7 and 5 amino acids deficiency in the insertion region compared with normal HsfA members. Eight StHsfs without amino acids insertion belong to class B, while one StHsf with 7 amino acids between HR-A and HR-B core belongs to class C. These Hsfs were subsequently classified into several subclasses according to the phylogenetic analysis.

All of the 27 putative potato *Hsfs* were renamed as *StHsf001* to *StHsf027* based on the order of class and subclass classification to distinguish them from some existing names such as *StHsf8, StHsf24, and StHsf30*. The representative information of the *StHsf* genes and their encoding proteins, containing the genomic length, CDS length, amino acids numbers, the theoretical molecular weight, the isoelectric point and the grand average of hydropathicity, was presented in Table 1. The variation range of protein length of the putative Hsfs were from 201 amino acids (StHsf026) to 501 residues (StHsf005), implying their structure difference and function diversity. The theoretical pI varying from 4.71 (StHsf014) to 9.58 (StHsf026) indicated that these Hsf proteins may exist and function in different regions of cells. The predicted results of grand average of hydropathicity of these deduced proteins revealed that they all belong to hydrophilic proteins.

**Table 1:** **The list of StHsf members identified**.

**Proposed name**	**Protein sequence number in Spud DB Potato Genomics Resources**	**Accession number in NCBI**	**Chr**	**Genomic length(bp)**	**CDS length (bp)**	**No.of amino acids**	**MW(KDa)**	**pI**	**GRAVY**	**Class**
StHsf001	PGSC0003DMP400005713	XP_006347585.1	chr03	5461	1479	492	54.3187	4.85	−0.440	A1b
StHsf002	PGSC0003DMP400026069	XP_006352886.1	chr08	5698	1497	498	55.7212	5.01	−0.671	A1c
StHsf003	PGSC0003DMP400046951	XP_006347336.1	chr06	4534	1470	489	55.2681	5.13	−0.594	A1e
StHsf004	PGSC0003DMP400014459	XP_006351581.1	chr08	2840	1062	353	40.7097	4.92	−0.659	A2
StHsf005	PGSC0003DMP400004805	XP_006341165.1	chr09	3361	1506	501	55.3798	4.80	−0.500	A3
StHsf006	PGSC0003DMP400048368	XP_006356094.1	chr03	5638	1212	403	46.2135	5.16	−0.770	A4a
StHsf007	PGSC0003DMP400030290	XP_006348918.1	chr07	1733	1254	417	47.5478	5.36	−0.741	A4b
StHsf008	PGSC0003DMP400049433	XP_006358092.1	chr02	6716	1227	408	46.2204	5.23	−0.764	A4c
StHsf009	PGSC0003DMP400008251	XP_006349912.1	chr12	4014	1437	478	53.4251	5.48	−0.699	A5
StHsf010	PGSC0003DMP400011443	XP_006363482.1	chr09	2541	1089	362	42.2633	5.46	−0.931	A6a
StHsf011	PGSC0003DMP400028424	XP_006350653.1	chr06	2119	1035	344	39.7640	5.03	−0.845	A6b
StHsf012	PGSC0003DMP400033606	XP_006349720.1	chr09	2554	1098	366	42.3105	5.45	−0.785	A7a
StHsf013	PGSC0003DMP400033608	XP_006349724.1	chr09	2521	1086	361	41.6117	5.30	−0.708	A7b
StHsf014	PGSC0003DMP400030540	XP_006340011.1	chr09	6966	1185	394	45.5392	4.71	−0.593	A8a
StHsf015	PGSC0003DMP400063465	N/A	chr02	1324	1236	411	46.9544	4.92	−0.809	A8b
StHsf016	PGSC0003DMP400065338	N/A	chr11	826	741	246	28.7078	9.30	−0.651	A8c
StHsf017	PGSC0003DMP400049438	N/A	chr02	1510	1023	340	38.4500	6.59	−0.909	A8d
StHsf018	PGSC0003DMP400055694	XP_006357708.1	chr07	2202	1086	361	41.7611	5.28	−0.825	A9
StHsf019	PGSC0003DMP400007176	XP_006339901.1	chr02	3544	909	302	33.2882	5.75	−0.754	B1
StHsf020	PGSC0003DMP400025228	XP_006353396.1	chr03	1205	1008	335	36.8417	4.84	−0.728	B2a
StHsf021	PGSC0003DMP400005485	XP_006358879.1	chr08	1651	954	317	35.0239	4.93	−0.472	B2b
StHsf022	PGSC0003DMP400047457	XP_006350178.1	chr04	2199	744	247	28.6956	8.68	−0.754	B3a
StHsf023	PGSC0003DMP400014364	XP_006351255.1	chr10	2284	735	244	28.3760	6.13	−0.857	B3b
StHsf024	PGSC0003DMP400014004	XP_006354868.1	chr04	1446	1119	372	42.3672	7.76	−0.714	B4a
StHsf025	PGSC0003DMP400056532	XP_006356390.1	chr11	2641	1125	374	44.1366	9.19	−0.580	B4b
StHsf026	PGSC0003DMP400051767	XP_006347051.1	chr02	2109	606	201	23.8534	9.58	−0.845	B5
StHsf027	PGSC0003DMP400000756	XP_006353456.1	chr12	1748	1107	368	40.9740	5.86	−0.614	C1

Chr, chromosome numbers; GRAVY, grand average of hydropathicity; MW, molecular weight; pI, isoelectric point; N/A, not applicable.

### Phylogenetic analysis of Hsfs in potato and other plant species

**Figure 1:**
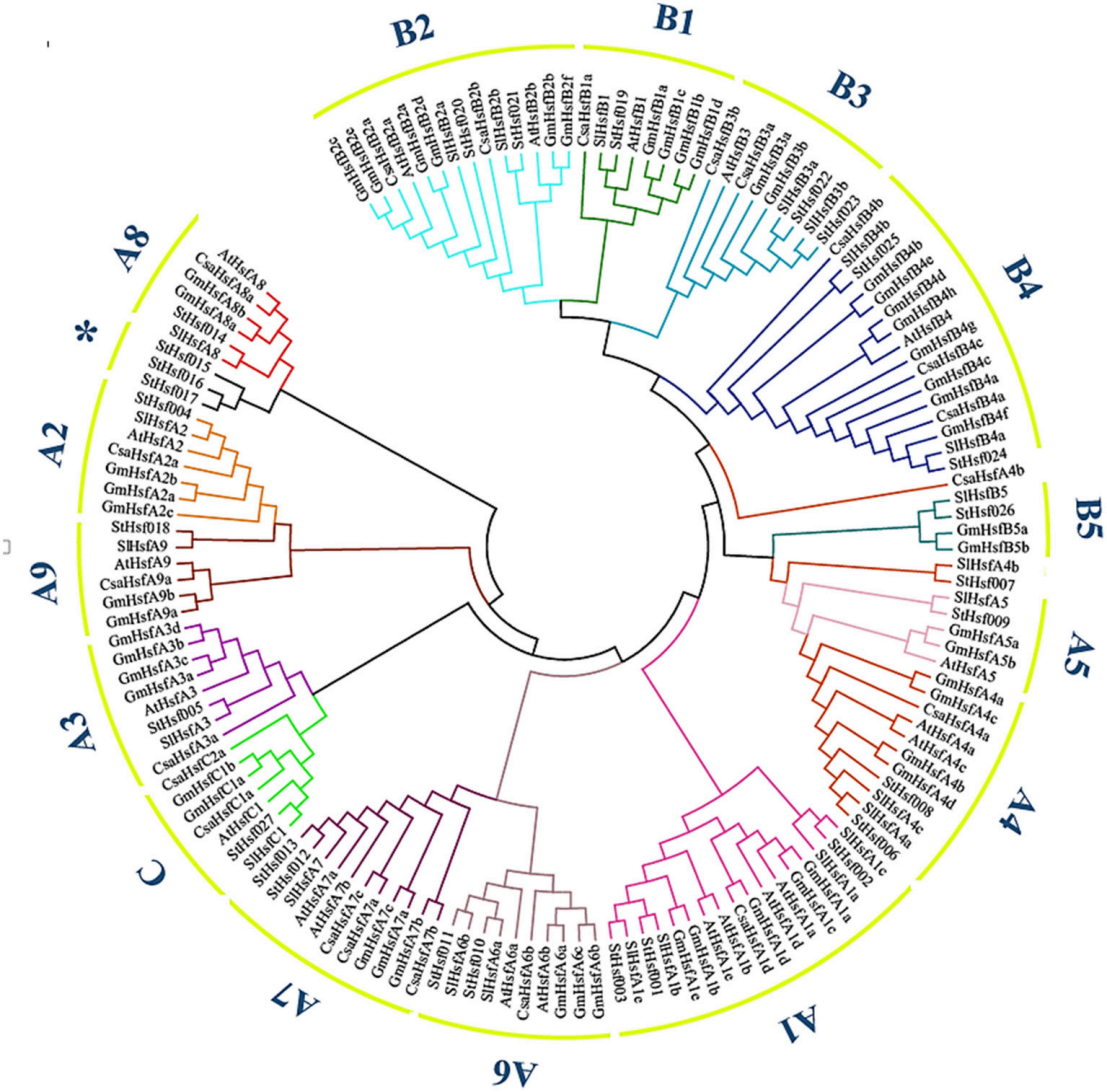
**The Neighbor-Joining phylogenetic tree of Hsf proteins from potato, Arabidopsis, tomato, cucumber and soybean**. The phylogenetic tree was constructed by Clustal X 2.1 and MEGA 4.0 software using the neighbour-joining option with 1000 bootstrap replicates. Branch lines in different colors represented different subgroups.

The phylogenetic analysis of potato helped to classify these Hsf members into several subclasses in comparison with the classification scheme of other species which have a close phylogenetic relationship with potato (Figure 1). One small branch marked with an asterisk was unique in potato and classified them into StHsfA8^*^ members because they have high similarity with other members of A8 subclass (Figure 1 and Table 1). In potato, class A and B members were further divided into A1–A9 and B1–B5 subgroups, respectively. Among these subgroups, HsfA1, HsfA4, HsfA6, HsfA7, HsfA8, HsfB2, HsfB3, and HsfB4 were each composed of more than two members; while class C and other subclasses each contained only one member (Figure 1 and Table 1).

The number of StHsfs belonging to each subclass was compared with that in other plant species where this family has been fully identified, such as *Arabidopsis* (*Arabidopsis thaliana*) (Nover et al., 2001), tomato (*Solanum lycopersicum*) (Baniwal et al., 2004), soybean (*Glycine max*) (Chung et al., 2013), rice (*Oryza sativa*) (Wang et al., 2009), maize (*Zea mays*) (Lin et al., 2011), poplar (*Populus trichocarpa*) (Wang et al., 2012), and pepper (*Capsicum annuum*) (Guo et al., 2015; Table 2). The composition of Hsfs in potato is similar to *Arabidopsis*, tomato, soybean, poplar and pepper (all of them are dicotyledonous plants), especially tomato and pepper which are another two kinds of solanaceae plants, but differs significantly from rice and maize, which are monocotyledon. *HsfA9, HsfB3*, and *HsfB5* are specific genes in dicotyledonous plants indicating that these types might appear after the differentiation of dicotyledonous and monocotyledonous plants. Another detectable difference between monocotyledonary and dicotyledonary Hsfs is that gene duplication in monocot develops a monocotyledon-specific group containing *C1a, C1b, C2a*, and *C2b* while in most dicotyledon only *C1* members exist, which is consistent with the result of Scharf et al. (2012).

**Table 2:** **Size of the Hsfs classes and subclasses in different plant species**.

	**Name**	***Arabidopsis thaliana***	***Solanum lycopersicum***	***Solanum tuberosum***	***Glycine max***	***Oryza sativa***	***Zea mays***	***Populus trichocarpa***	***Capsicum annuum***
A	HsfA1	4	4	3	5	1	2	3	3
	HsfA2	1	1	1	3	3	2	1	1
	HsfA3	1	1	1	4	1	1	1	1
	HsfA4	2	3	3	4	2	3	3	3
	HsfA5	1	1	1	2	1	1	2	1
	HsfA6	2	2	2	3	2	2	2	3
	HsfA7	2	1	2	3	2	2	2	0
	HsfA8	1	1	4	2	1	2	2	1
	HsfA9	1	1	1	2	0	0	1	4
B	HsfB1	1	1	1	4	1	2	1	1
	HsfB2	2	2	2	6	3	4	3	2
	HsfB3	1	2	2	2	0	0	2	2
	HsfB4	1	2	2	8	4	1	4	1
	HsfB5	0	1	1	2	0	0	2	1
C	HsfC1	1	1	1	2	2	2	1	1
	HsfC2	0	0	0	0	2	1	0	0
	Total	21	24	27	52	25	25	30	25

### Chromosomal distribution of *StHsf* genes

**Figure 2:**
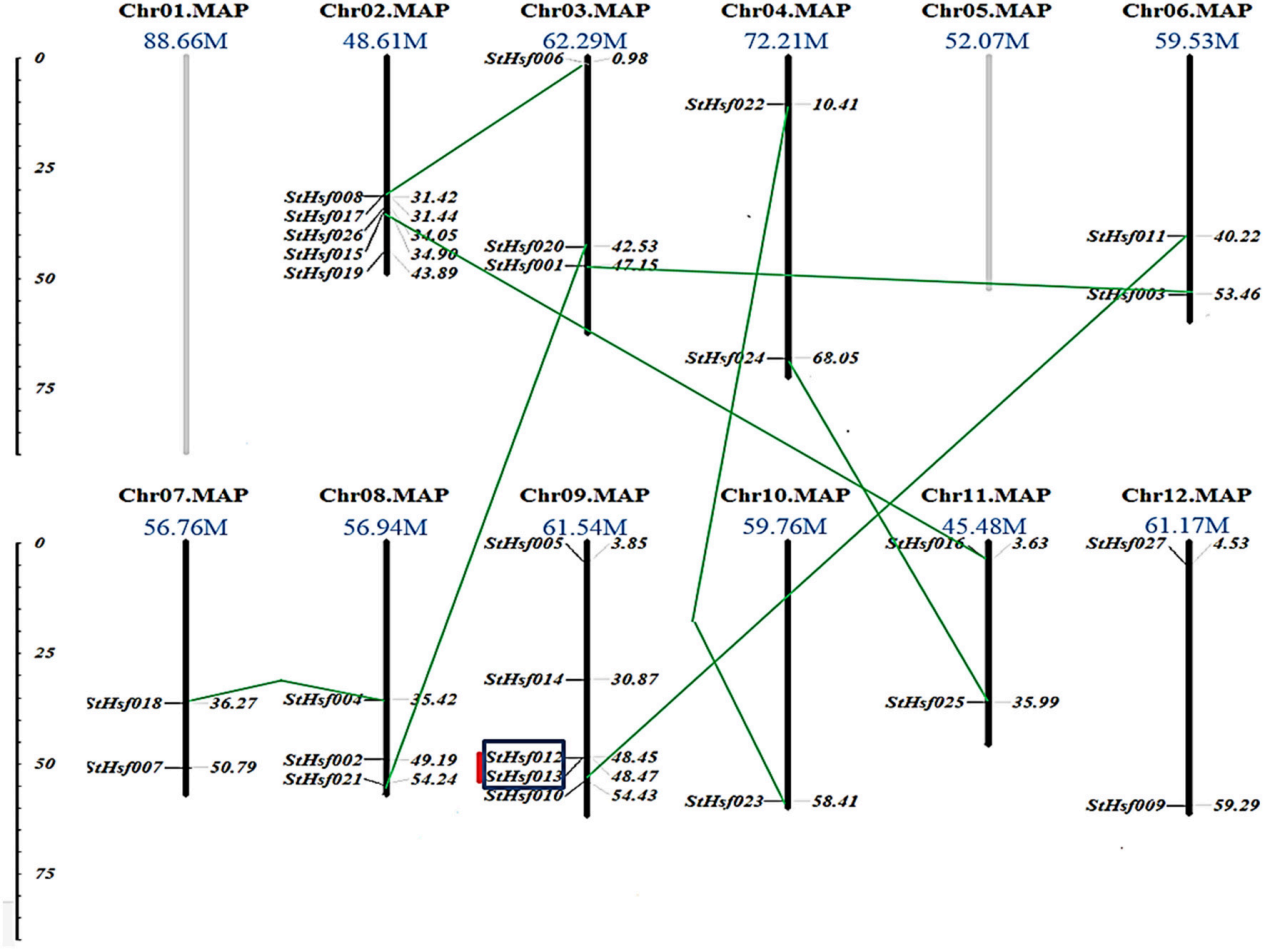
**Chromosomal locations of *StHsf* genes**. Chromosome numbers and length are represented at the top of each chromosome. Paralogous genes are linked by green lines. Genes belonging to clusters are indicated in black box. Tandem duplications are marked by red lines.

In order to investigate the distribution of *Hsf* genes on different chromosomes in potato, the location of 27 deduced *StHsfs* were identified according to the information of potato genome database on Spud DB Potato Genomics Resources. These *StHsfs* could be mapped to 10 of the 12 potato chromosomes, with no *StHsf* found on chromosome 1 and chromosome 5 (Figure 2). They unevenly distributed across the chromosomes of the potato genome: Chr10 contains only one *StHsf*, while relatively high densities of *StHsf* genes were discovered on Chr02 and Chr09 (5 *StHsfs* respectively). Most chromosomes contain two (Chr04, Chr06, Chr07, Chr11, and Chr12) or three *StHsfs* (Chr03 and Chr08).

**Figure 3:**
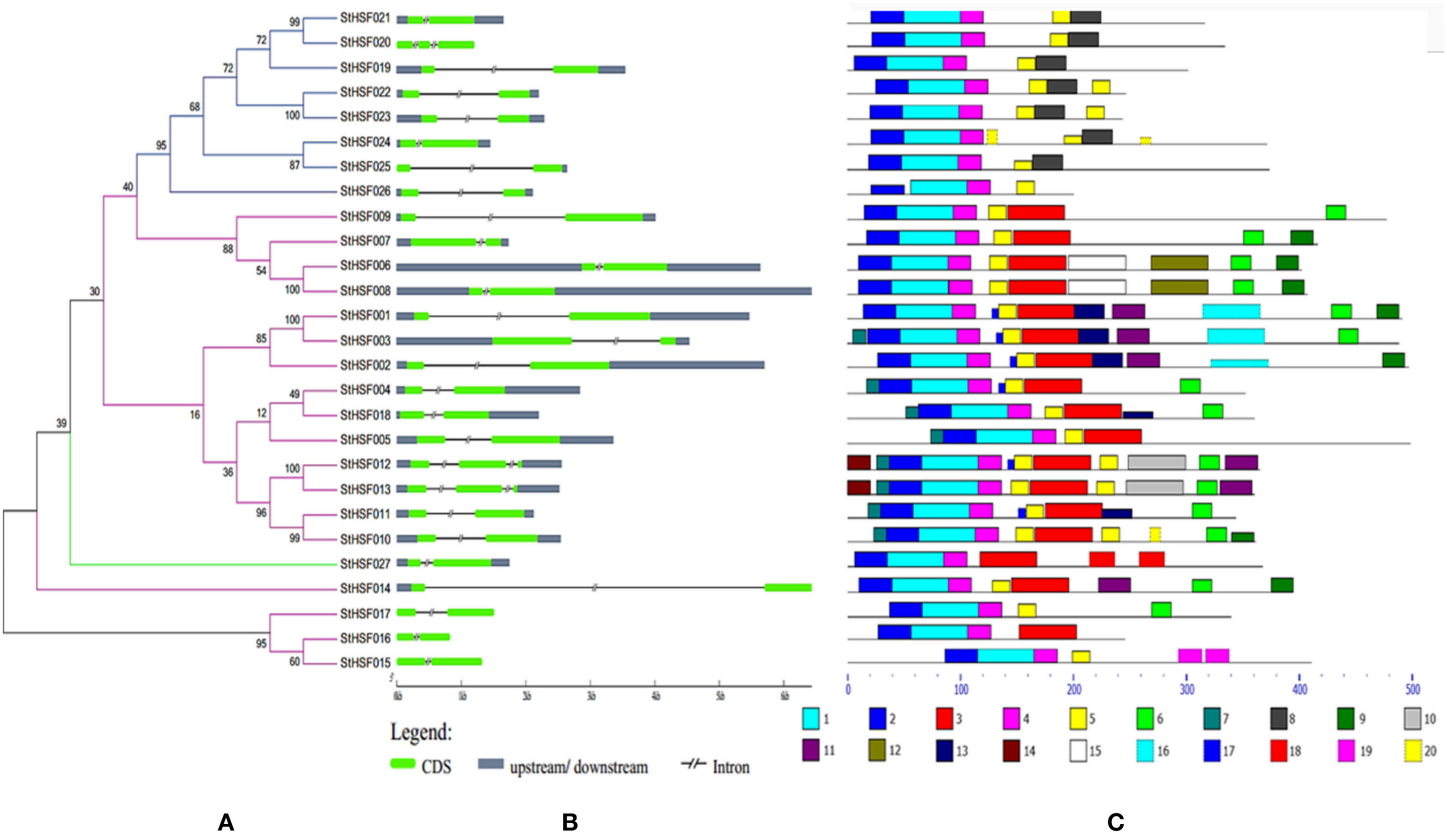
**Evolutionary relationships (A), gene structures (B) and functional motifs (C) of StHsfs. (A)** The phylogenetic tree was constructed by Clustal X 2.1 and MEGA 4.0 software using the neighbour-joining method with 1000 bootstrap replicates. Subtrees branch lines in different colors represent different Hsf classes. **(B)** The exon/intron distribution of corresponding *StHsf* genes was detected by comparing these predicted coding sequences (CDS) with their corresponding genomic sequences using GSDS online (http://gsds.cbi.pku.edu.cn). The green boxes represent CDS; the blue boxes indicate upstream or downstream; the discontinuous lines refer to introns of these genes. **(C)** The motif composition related to each StHsf protein is displayed on the right-hand side. The motifs, numbered 1–20, are displayed in different colored boxes. The sequence information for each motif is presented in Table 3.

All of the 27 *StHsfs* were single copy genes. According to the phylogenetic tree of deduced StHsf sequences (Figure 3A), we linked the paralogous pairs of *StHsfs*, and found a total of 9 pairs of paralogous *StHsfs*: *StHsf001/StHsf003, StHsf006/StHsf008, StHsf004/StHsf018, StHsf010/StHsf011, StHsf012/StHsf013, StHsf015/StHsf016, StHsf020/StHsf021, StHsf022/StHsf023*, and *StHsf024/StHsf025* (Figure 2). Tandem duplications of paralogous genes, defined as two paralogs separated by less than five average-gene-length in the same chromosome, have been suggested to be the main cause for gene family expansion in plants (Yuan et al., 2015). With that definition, *StHsf012* and *StHsf013* on chromosome 9 (location: 48.449647Mb/48.466395Mb) was found to be a pair of tandem duplications. A chromosome region containing two or more genes within 200 kb can be defined as a gene cluster (Holub, 2001; Zhang et al., 2015). Therefore, *StHsf012* and *StHsf013* also belong to a gene cluster.

### Phylogenetic analysis, exon-intron organization and conserved motifs of the StHsf family

The phylogenetic tree created by the alignment of these full-length StHsf sequences illustrated the evolution relationships of Hsf members in potato (Figure 3A). These StHsfs are classified into A, B and C classes as described above. The class A members develop into three smaller groups in comparison with class B and class C Hsfs which respectively form into a single group. The class C member (StHsf027) has an even closer evolution relationship with class A members than class B members implying that class C Hsf probably evolved from class A Hsfs.

The gene structure diagram depicted that most *StHsf* genes have one intron, while 3 genes (*StHsf012, StHsf013*, and *StHsf022*) contain two introns (Figure 3B). The length of intron exhibited certain degrees of variation, which is similar to that in other plants like *Arabidopsis* (Nover et al., 2001) and tomato (Baniwal et al., 2004). The exon-intron structure of most homologous gene pairs shared the similarity (*StHsf006/StHsf008, StHsf004/StHsf018, StHsf010/StHsf011, StHsf012/StHsf013, StHsf015/StHsf016*); while some homologous genes had several differences in intron numbers (*StHsf020/StHsf021*), intron length (*StHsf022/StHsf023, StHsf024/StHsf025*), and intron position (*StHsf001/StHsf003*).

Twenty conserved motifs of the deduced StHsf proteins were predicted by MEME motif detection software to reveal the conservation and diversification of these proteins in structure and function (Figure 3C). The details of the 20 putative motifs were shown in Table 3. As shown in Figure 3C, motif 1 was connected with motif 2 and motif 4 closely. This joint cluster of motifs 1, 2, and 4 represents the most conservative domain (DBD) existing in all 27 StHsf proteins. Besides, motifs 3, 5, and 8 were also conserved domains in StHsfs. Motif 5 and motif 3 were inferred as the OD region of StHsfAs, and motif 5 and motif 8 were considered as the OD of StHsfBs according to the conversed hydrophobic positions of HR-A and HR-B in StHsfs (Supplementary Figure S2). Moreover, StHsf members within the same subgroups were generally found to share a common motif organization. A unique motif, named as motif 6, was detected in 15 of 18 Hsfs. This motif was close to the C terminal and was deduced to be an AHA motif. These analyses suggested that most of the closely related members in the phylogenetic tree shared common motifs within the same group and possessed similar exon-intron arrangements.

**Table 3:** **Analysis and distribution of conserved motifs in potato StHsfs**.

**Motif**	***E*-value**	**Width**	**Best possible match**
1	1.8e-945	50	FIVWDPPEFARDLLPKYFKHNNFSSFVRQLNTYGFRKIDPDRWEFANEWF
2	1.0e-360	29	PPPFLTKTYEMVDDPSTDHIISWNRNGTS
3	2.4e-218	50	LMMELVKLRQHQQATDHYMQTMTQRLQATEQRQQQMMSFLAKAMQNPGFV
4	1.9e-184	20	LRGQKHLLCNIHRRKTWHSH
5	1.7e-050	15	RIGYEEEIERLRRDK
6	1.0e-024	17	NDIFWEQLLTENPICGD
7	9.3e-016	11	PQPMEGLHDIG
8	1.2e-013	26	MLSSELTHMKKLCNDIIYFMSNYVKP
9	1.8e-014	19	WWNLKHMHHLTEQMGHLTP
10	2.3e-012	50	HVGGFSHYIKSEPLEFGEANGFQVSELEALALEMQGFGRARKDQQEEYTI
11	1.3e-011	28	CVDTLADGQIVRYQPIMHEAAKWINQCI
12	5.7e-007	50	AGMRQNCSIDLDESISCADSPAISYPQLNVDVGPKASGIDMNSEPNGNTT
13	3.3e-003	26	AQLVHQQNDNNRRIPGMNKKRRIPQQ
14	1.3e-001	20	MMNQLYSVKEEFPGSSSGGG
15	2.2e-001	50	MPQLQMNDRKRRFPGNSCLYNETGLEDMRGISSRALTRENMDPTSLLTMN
16	2.8e-001	50	QPHLMSDSGFPFNSCLSIIPEIQYSPTVVPGQAKIPQFPEADALNSQADH
17	4.6e-001	6	ACVEVG
18	7.6e+000	22	RSPDANFDKDAFCQSSPSSGTP
19	1.3e+000	20	KKRKMQVVEELEGDNEGRKK
20	1.e+001	8	MEPHHYYH

### *StHsfs* expression in different organs and tissues

**Figure 4:**
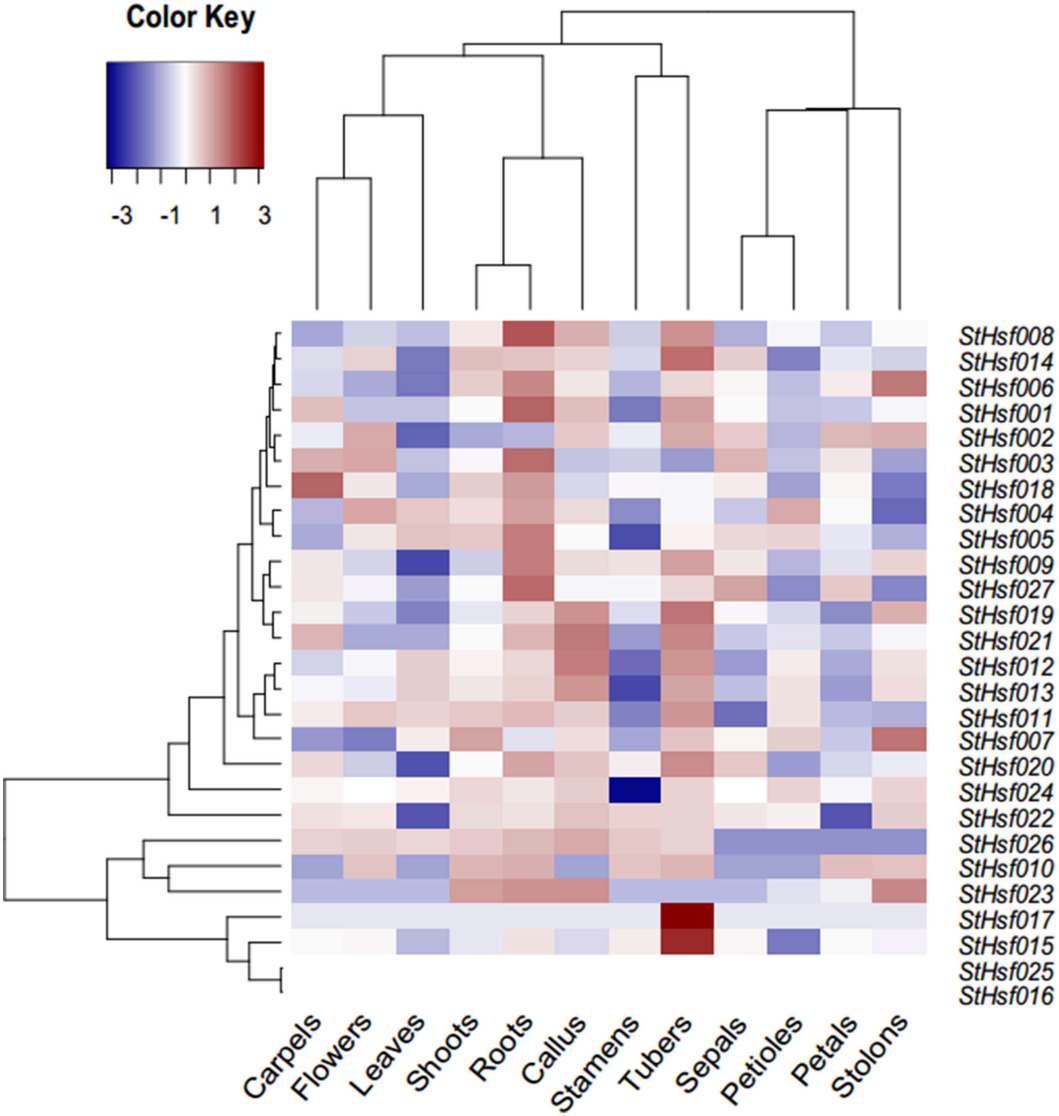
**Expression profiles of the *StHsf* genes in different potato organs and tissues**.

A *StHsf* expression heatmap of 12 different organs and tissues, which established using RNA-seq data (Supplementary Table S2), showed a higher expression of most genes in tubers, callus, shoots and roots than in other tissues, such as leaves, stamens and petals (Figure 4). Some *StHsf* genes shared a highly similar expression profile in various potato tissues. For example, *StHsf012* and *StHsf013*, belonging to *HsfA7*, were highly expressed in tubers and callus while lowly expressed in stamens, petals, sepals and carpels. Other members of B group, such as *StHsf019, StHsf020, StHsf021, StHsf022, StHsf023*, and *StHsf024*, exhibited high levels of expression in vegetative organs, such as shoots, roots, tubers, stolons and callus, suggesting an involvement of class B members in plant vegetative growth. Some members of class A including *StHsf002, StHsf003* and class C member *StHsf027*, were characterized by high expression amounts in vegetative organs and also in sepals, petals, carpals and the whole flowers than other *StHsf* members. Within the *HsfA1* group, *StHsf001* was highly expressed in roots, tubers and carpels while *StHsf002* had high expression in tubers, stolons, flowers, petals, sepals and callus and the expression of *StHsf003* were high in roots, flowers, carpels and sepals. This analysis indicated that the plants have adaptation reaction to harmful environment through compensation of *Hsf* genes to lighten the menace action of adversity.

### Promoter and qRT-PCR analyses of *StHsf* genes on stress responses

**Figure 5:**
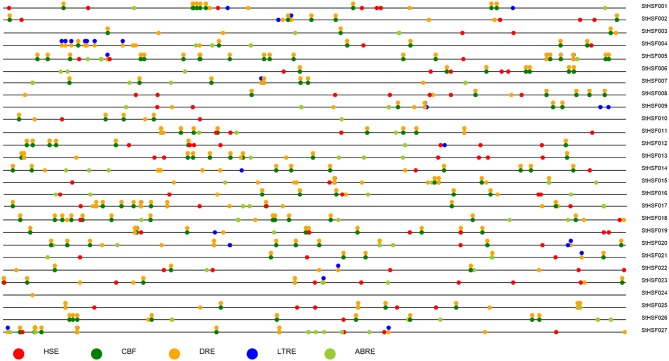
**Promoter analysis of 27 deduced *StHsfs***. The circles in different colors represent different stress response elements of the −2 Kb 5′' upstream region of 27 *StHsfs*.

Response elements of *StHsf* genes' promoters, including Heat Stress Element (HSE), C-Repeat Binding Factor (CBF), ABA Responsive Element (ABRE), Dehydration-Responsive Element (DRE), and Low Temperature Responsive Element (LTRE) were displayed in Figure 5. The promoter analyses showed that nearly all of these genes had multiple HSEs, CBFs, ABREs, DREs, and LTREs in their promoter regions except *StHsf024* whose promoter sequence has not been completed. All these genes had multiple DREs suggesting that they are responsive to drought stress. Some genes are lacking of one or two response elements, such as *StHsf007* without HSE and *StHsf008* without LTRE, which implied that the expression of these genes might be weakly influenced by the corresponding stresses or not be directly induced by these stresses. In general, the promoter analysis suggested that all of *StHsf* genes should be responsive to various abiotic stresses.

**Figure 6:**
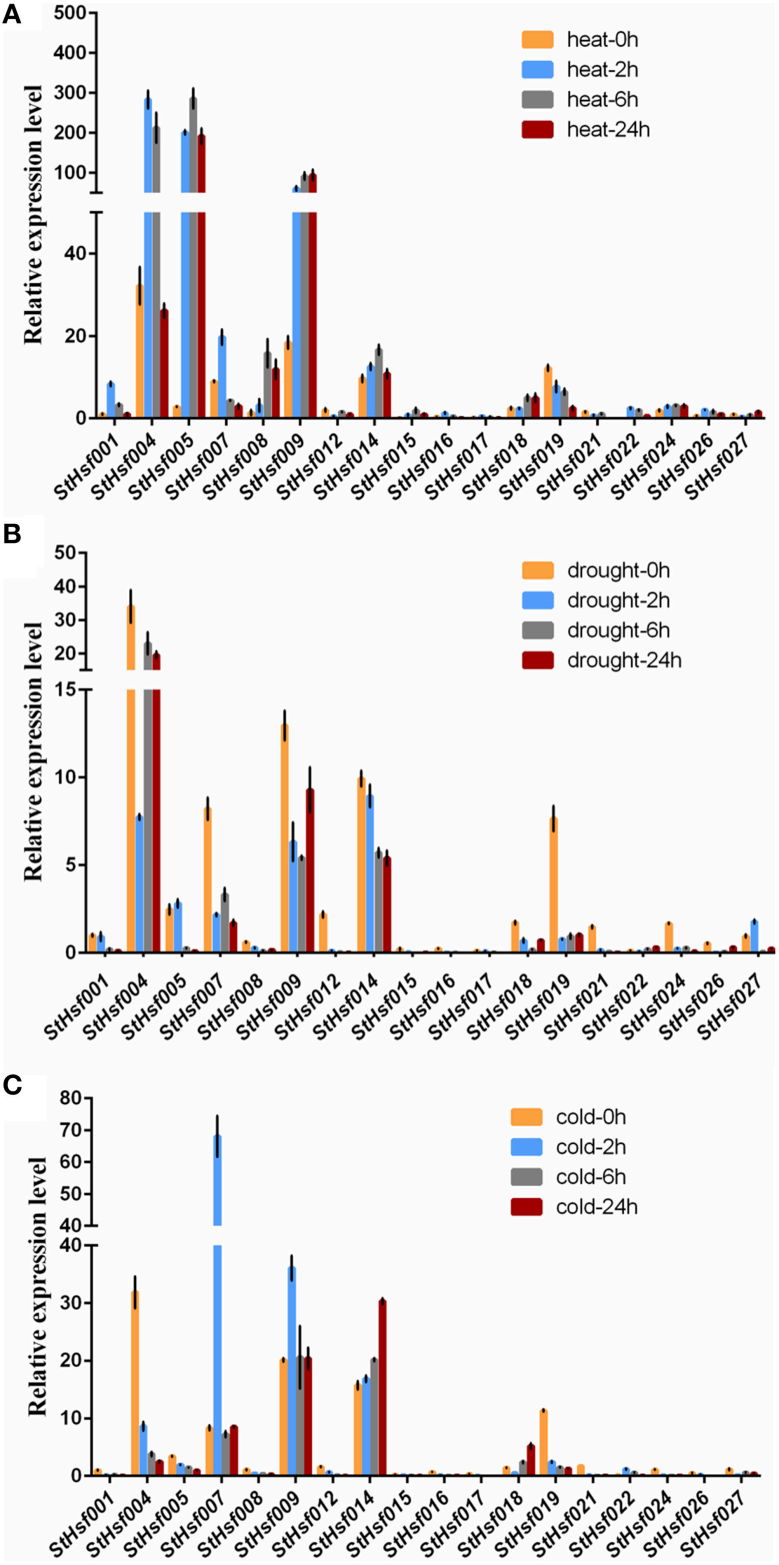
**Relative mRNA abundance of *StHsf* members in response to heat (A), drought (B) and cold stress (C) in the leaves**.

To validate this hypothesis, qRT-PCR was used to determine the expression profiles of *StHsf001, 004, 005, 007, 008, 009, 012, 014, 015, 016, 017, 018, 019, 021, 022, 024, 026*, and *027* genes in leaves of potato plants subjected to heat, drought and cold stresses (Figure 6). These *StHsf* genes were selected from all subgroups and the primers were presented in Supplementary Table S1. The heat, drought and cold responsiveness of these genes was examined in the leaves of 1-month-old plants with short- (2 h and 6 h) and long-term (24 h) stress treatments. In order to clarify which genes play the major role during different stress conditions as a whole, the expression of *StHsf001* at 0 h under the corresponding stress was set to 1 and the expression of other genes in different stages of treatment were compared with that. Generally, the expression levels of *StHsf004, StHsf005, StHsf007, StHsf009*, and *StHsf014* were all higher than other genes during different stresses. Specifically, *StHsf004, StHsf005*, and *StHsf009* became predominant *StHsf* transcripts during heat stress, especially *StHsf004* and *StHsf005* whose expression levels were approximately 200~300 times higher than that of the other members (Figure 6A); while *StHsf004, StHsf007, StHsf009*, and *StHsf014* all played a leading role under drought stress (Figure 6B) and cold stress (Figure 6C). In addition, constitutive expression of *StHsf004, StHsf005, StHsf007*, and *StHsf012* was observed before stress treatments, which is in accordance with the expression heatmap of the *StHsf* genes in potato leaves (Figure 4).

This qRT-PCR data was also used to analyze the expression changes of each gene in heat, drought and cold stress treatments separately (Supplementary Figure S3). In general, the expression amounts of selected genes in each stress treatment had fluctuated over these 24 h. The expression levels of most genes began to show dramatic changes at 2 or 6 h, which indicated that *StHsfs* are sensitive to abiotic stresses. In particular, during heat stress, the expression of most class A members in leaves was up-regulated. For instance, the transcript levels of *StHsf001, StHsf004, StHsf007, StHsf016*, and *StHsf017* were up-regulated and reached the peak level at 2 h, and the expression of *StHsf005, StHsf008, StHsf009, StHsf014, StHsf015*, and *StHsf018* was peaked at 6 or 24 h. However, the mRNA level of *StHsf012* was down-regulated with a 2 h heat treatment but an increase was observed in the 6 h treatment. Heat up-regulation was also observed in most *HsfB* members with 2 h treatment, such as *StHsf022, StHsf024*, and StHsf026, especially *StHsf022* whose expression amount at 2 h soared to about 50-fold of expression level at 0 h. Conversely, the gene expression of *StHsf019* declined gradually and dramatically under heat stress. The expression level of *StHsf027*, the class C member, declined in the 2 h treatment but increased at 6 h treatment.

Although the major role of Hsfs is well known to be the regulation of heat-responsive genes involved in heat acclimatization, it was also of interest to detect whether this family is involved in response to other major abiotic stresses such as drought and cold stresses. The expression of these genes was induced by drought stress as the same as heat stress but the expression pattern of each gene was different from that in heat condition. More exactly, the expression levels of most of these genes mediated by drought stress witnessed a downward trend from 0 to 2 h onwards, which was in contrast to the expression pattern under heat treatment. Some genes such as *StHsf005* and *StHsf027*, however, were up-regulated by drought transiently. As for cold stress, these genes presented a different expression pattern from heat and drought. Apart from *StHsf007, StHsf009, StHsf014, StHsf018*, and *StHsf022*, whose expression amounts had an increase trend, the other selected genes dipped steadily.

### Construction of co-expression network and identification of *StHsf*-related genes

**Figure 7:**
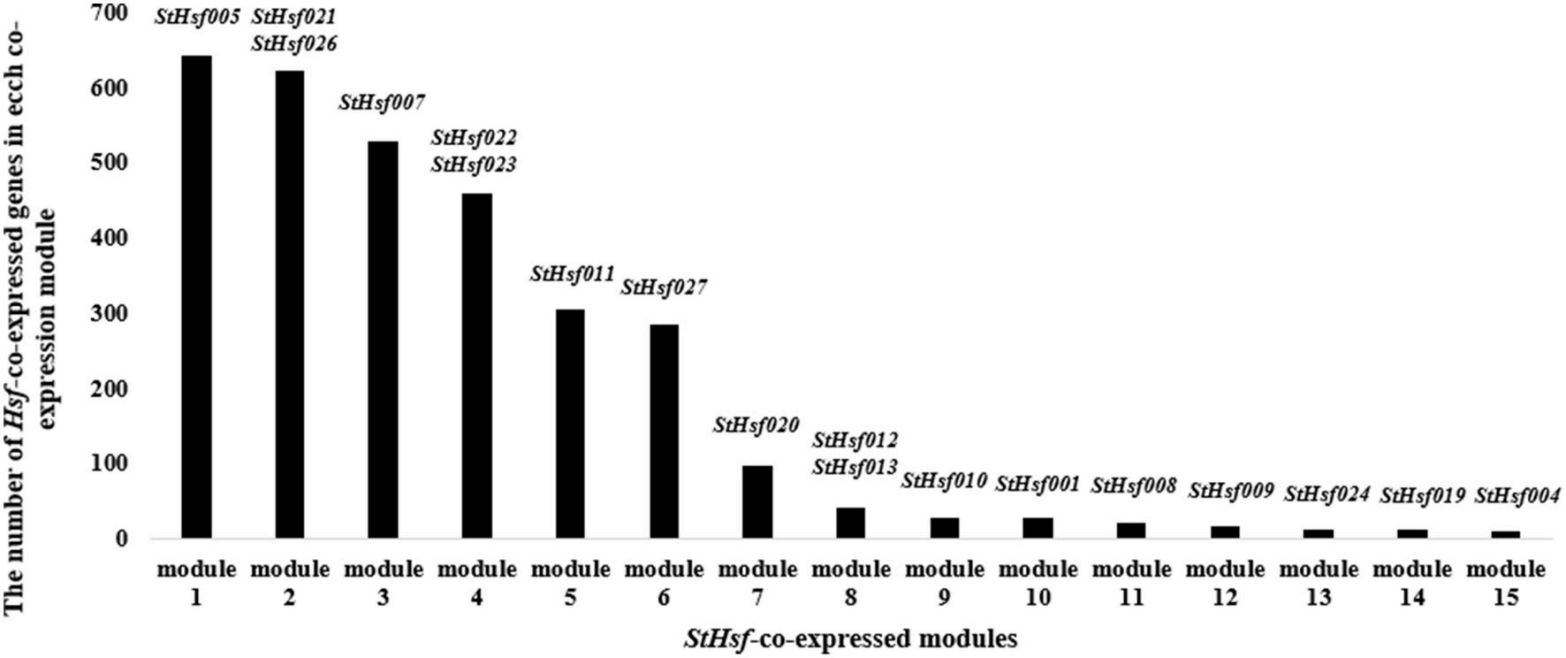
**The number of *StHsf*-co-expressed genes in each co-expression module**. The modules are numbered according to the gene numbers. *StHsf* genes involved in the corresponding module are placed at the top of each column.

In order to delve into the potential regulatory relationship between *StHsfs* and their related genes, a co-expression network was constructed using WGCNA according to the RNAseq data. As shown in Figure 7, 15 modules containing different *StHsfs* and their co-expressed genes were formed based on the correlation of biological function among genes. Some *StHsfs* (*StHsf002, StHsf003, StHsf006, StHsf014, StHsf015, StHsf016, StHsf017, StHsf018*, and *StHsf025*) were not shown in Figure 7 because of their low expression levels in RNAseq data. Module 1, which contained *StHsf005* and other 642 related gene members, was the largest module among the 15 modules; while module 15, containing only 10 genes, was the smallest module (Figure 7). Because module 1 contained more genes than other modules, we hypothesize that module 1 is involved in more biological functions than other modules.

**Figure 8:**
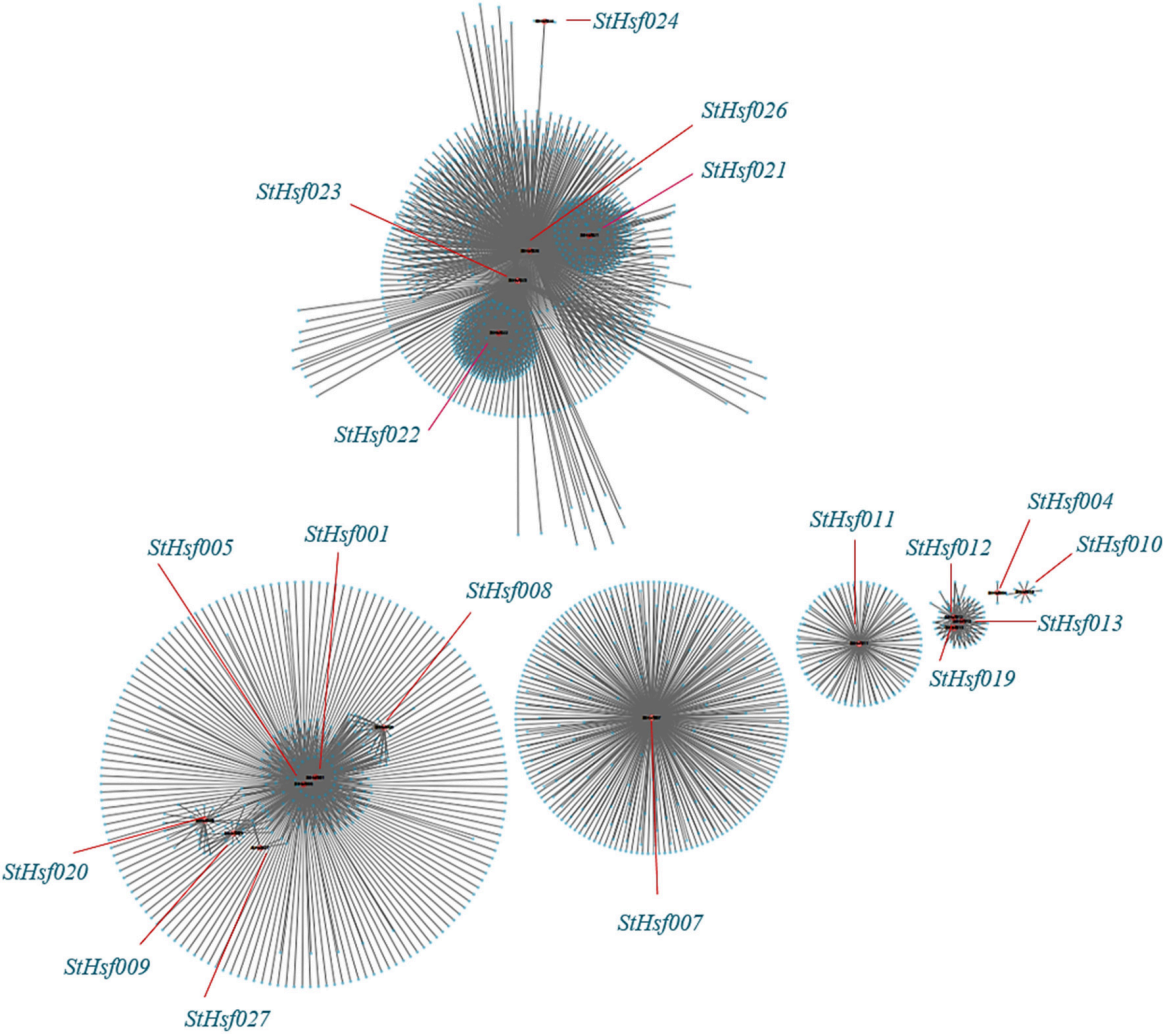
**Co-expression network of *Hsfs* and their co-expressed genes in potato**. TOM (Topological overlap matrix) value is 0.07. Nodes represent *StHsf* and co-expressed genes in potato, edges indicate pairwise correlation constructed by WGCNA. Red circles indicate *Hsfs*, blue circles represent *Hsps*. The network was created using Cytoscape (see Materials and Methods). The length of the line segment doesn't stand for the closeness of the relationship between *StHsf* and each gene; it is simply for distinguishing different genes.

The co-expression network of *Hsfs* and their related genes in potato was created by Cytoscape with TOM (Topological overlap matrix) value 0.07 (Figure 8). The genes in a module were formed into a circle with *StHsf* as a center (red points) and other related genes around (blue points). The sequence numbers and potential functions of these genes related to *StHsfs* in Spud DB Potato Genomics Resources were provided in Supplementary Table S3. Proteins, encoded by these genes, are not only StHsps like Heat shock cognate 70 kDa protein (PGSC0003DMT400001180) and 10 kDa chaperonin (PGSC0003DMT400060280), but also other genes with a variety of functions, such as DELLA protein (PGSC0003DMT400049445), MAPK (PGSC0003DMT400077272) and MAPKKK (PGSC0003DMT400057171) in the module 3 which contained *StHsf007*. Except the relationship between *StHsfs* and their correlated genes, there also existed direct or indirect interactions between *StHsfs*, such as *StHsf001, StHsf005, StHsf008, StHsf009, StHsf020*, and *StHsf027* (Figure 8). These results provide some basis for further study of function mechanism of StHsfs.

## Discussion

### Hsfs in the same class and subclass share similar gene structures, conserved motifs and regulatory functions

Hsfs, which exist extensively in all plant species, have been considered as a sort of important regulators in response to abiotic stresses based on the researches of Hsfs in some species like *Arabidopsis* (Nover et al., 2001), tomato (Mishra et al., 2002), apple (Giorno et al., 2012), and cabbage (Ma et al., 2014). Nevertheless, few researches on *Hsfs* in potato have been reported. As the potato genome sequence has been completed, 27 *Hsf* genes from *Solanum tuberosum* were identified and analyzed in phylogenetic relationships, gene structures, conserved motifs, chromosomal locations, promoters and expression patterns. These Hsf members which belong to the same class and subclass had a close evolutionary relationship and shared a similar exon-intron structure and motif composition, implying their similar regulatory functions.

Orthologous genes which exist in different species from a common ancestor may or may not have the same function; while paralogous genes within a single species that created by gene duplication may evolve new functions related to the original genes (Thornton and DeSalle, 2000; Guo et al., 2008). Therefore, gene orthology analysis can be used as a preliminary method to explore the function of candidate genes (Wang et al., 2014). HsfA1a has been reported as an irreplaceable master transcription factor of induced heat tolerance in tomato but no similar Hsfs were found in *Arabidopsis* (Lohmann et al., 2004; Von Koskull-Döring et al., 2007). In potato, HsfA1a was not identified according to Figure 1. Therefore, further function analysis of HsfA1 in potato is required to justify whether there exists a master regulator as in tomato. HsfB1, which was considered to enhance the activity of recruiting histone acetyltransferase (HAC1) by collaborating with tomato HsfA1a, has similar functions in tobacco and soybean but acts as a repressor of HsfAs in *Arabidopsis* (Von Koskull-Döring et al., 2007). Further investigation of the HsfB1 sequences of tomato, *Arabidopsis* and potato exposed that the histone-like motif (GRGKMMK) in tomato HsfB1 is also found in potato but is changed by GSRMTETK in Arabidopsis, indicating that potato HsfB1 probably has a similar function as tomato HsfB1 (Supplementary S1; Bharti et al., 2004; Von Koskull-Döring et al., 2007; Zhu et al., 2012).

From the paralogous genes analysis of potato, class A Hsf members were observed to develop into several smaller groups whereas class B and class C members just formed a single group respectively, suggesting that the class A Hsfs might be derived from different gene ancestors compared with class B and class C Hsfs. Therefore, the class A was reported as a paraphyletic group, while the class B and class C were regarded as a monophyletic group (Scharf et al., 2012). Gene duplication and gene deficiency events usually occurred at different stage of plants evolution resulting in Hsfs diversity (Lin et al., 2006). One pair of paralogous StHsf genes, StHsf012/StHsf013, appeared to have undergone tandem duplication, which suggested that they probably derived from a same gene ancestor and have the same function. *StHsf015, StHsf016*, and *StHsf017*, which are unique genes in potato, might have mutation in the process of evolution. Closer observation of sequences in DBD and insertion area between HR-A and HR-B reveals that mutation such as replacement or deficiency of some amino acids may cause changes of their structure and function. For example, the α_3_ conserved sequence –RQLN- was replace by –CQLN-, -YQLN-, and -YQLN- in StHsf015, StHsf016 and StHsf017, respectively (Supplementary Figure S1). To our knowledge, R is a kind of positively charged amino acid while C and Y are polar amino acids without charge, which might have an impact on their function. Also, the OD regions of StHsf015, StHsf016, and StHsf017 were found to be incomplete compared with the normal OD domain, which offered another possibility of their changed function (Figure 3C and Supplementary Figure S2).

The number of potato Hsf members is close to plant species like Arabidopsis, tomato, rice, maize, poplar, and pepper in comparison with soybean. This probably results from the double duplications of genome in soybean but only a single replication in other plants during evolution (Blanc et al., 2000; Schlueter et al., 2004). Several Hsfs are specific to monocots or dicots. For example, the Hsf members of A9, B3, and B5 are restricted to dicots, and C2 are characteristic of monocots, suggesting the evolution of these subclasses after the divergence of monocots and dicots (Li et al., 2014).

Transcriptional activity of HsfA normally depends on the AHA motif in the C-terminal (Bharti et al., 2000). However, not all HsfA members contain this motif like StHsf005 (HsfA3) (Figure 3C). It was confirmed that HsfAs without an AHA motif could be activated by forming hetero-polymers with other HsfAs (Guo et al., 2008; Li et al., 2014). Most of the class B and C Hsfs do not possess the transcription activation ability like class A Hsfs due to the deficiency of AHA motif in their CTADs. Instead, the class B Hsf members are characterized with a tetrapeptide -LFGV- in their C-terminal region, which is proposed to act as a repressor motif in the transcription machinery (Ikeda and Ohme-Takagi, 2009). This tetrapeptide can be found in StHsf019, StHsf021, StHsf022, StHsf023, and StHsf024 (Supplementary S1), indicating the probable repression role of these five Hsfs in potato.

### Hsf members play crucial roles in potato growth and development and in response to intricate abiotic stresses

According to *Hsf* genes discovered so far, in-depth researches and new technologies enable the identification of more *Hsf* genes in various organisms. Considering the important roles that Hsfs play during plant development and in response to various stresses, it is not surprising that we identified so many Hsf family members in potato. The present research confirmed the involvement of Hsfs in tolerance to various stresses in potato, which is in agreement of the previous studies of Hsfs in *Arabidopsis*, rice, tomato and any other species (Mishra et al., 2002; Guo et al., 2008; Chauhan et al., 2011; Hahn et al., 2011; Yoshida et al., 2011).

Expression patterns analysis of all predicted *Hsf* members in 12 different potato organs or tissues uncovered that *StHsf* genes in the same group have a similar expression profile in potato tissues, implying that they may participate in a similar developmental process or regulatory pathway. Take the expression of *StHsf012* and *StHsf013* as an example, they were lowly expressed in the organs related to flower, suggesting that they may not participate in the regulation of flower development under non-stress condition. *HsfA9*, which has been reported in *Arabidopsis* to specifically express in seeds (Von Koskull-Döring et al., 2007), also expressed in carpels, roots and shoots of potato. This results indicated that *Hsf* members have different expression patterns in different plant species. All these *Hsfs* perform their own functions in different organs and tissues to make sure the normal growth and development of plants. Although some *Hsfs* expressed lowly in certain tissues, this does not mean that they are non-functional in these tissues. The expression of some *Hsfs* need to be induced by different stresses.

The constitutively expressed members (e.g. *StHsf004, StHsf007*, and *StHsf012*) expressed at relatively higher levels (Figure 6), which fits with the expression results of StHsfs in leaves as shown in Figure 4. In tomato, *HsfA1* was found to be a constitutively expressed gene and *HsfA2* was an induced up-regulated gene (Nishizawa et al., 2006; Von Koskull-Döring et al., 2007). In potato, however, the expression of *HsfA1b* (*StHsf001*) induced by heat stress treatment and *HsfA2* (*StHsf004*) constitutively expressed under non-stress condition and up-regulated by heat stress (Figure 6), which is opposite to the situation in tomato. The results of Liu and Charng (2013) have demonstrated that the function of HsfA1s can be replaced by HsfA2 in the absence of HsfA1s because of their high degree of sequence homology. But whether HsfA2 could perform the similar functions as HsfA1s in the low expression of HsfA1s needs further study. *StHsf004, StHsf005*, and *StHsf009*, belonging to A2, A3, A5 group, were the main StHsfA members that were up-regulated at a very high level during heat stress. Other class A genes that were up-regulated by heat were A4 and A8 group members (*StHsf007, StHsf008*, and *StHsf014*), but they were expressed at a low level under heat stress, compared with the A2, A3, and A5 groups. In terms of class B of the StHsf family, not all *StHsfB* members were down-regulated by heat; actually, the expression levels of the *StHsf022, StHsf024*, and *StHsf026*, which are B3, B4 and B5 members, increased after an initial induction. In class C, *StHsf027* was constitutively expressed in most organs. In leaves, the expression level of the C group was even higher than those of the A1 group and was up-regulated after a temporary descent with heat stress treatment.

The promoter analysis showed that most StHsfs contain multiple response elements in their promoter regions (Figure 5), presumably suggesting that these StHsfs could be transcriptionally activated by the combination of response elements and some stress-induced tans-acting factors. For example, Chen et al. (2010) has demonstrated that DREB2C can trans-activate the DRE-dependent transcription of HsfA3 by preferentially binding to the distal DRE2 located in the HsfA3 promoter under heat stress. *StHsf007*, which is lacking of HSE in its promoter, had an up-regulated expression in heat treatment (Figure 6A). One possibility is that under heat stress, some trans-acting factors like DREB2C might combine with the DRE located in the *StHsf007* promoter to activate the expression of *StHsf007*. However, the gene expression is a complex biological process and not only modulated by transcriptional regulation which is also a complicated process. The exact mechanism of gene expression needs further research.

The promoter analysis of *StHsfs* and a number of studies about *Hsf* gene family have provided evidence of their function to other abiotic stresses, reflecting that Hsfs are crucial regulators in the regulation of various stress responses (Swindell et al., 2007; Hu et al., 2009; Zhang et al., 2015). In respect to the function of HsfA3, it was reported to be induced by drought stress and was considered as a drought signal molecular in *Arabidopsis* (Sakuma et al., 2006; Von Koskull-Döring et al., 2007); while there is no considerable change of the expression of *StHsfA3* (*StHsf005*) after drought treatment in potato, which is similar to *HsfA3* in tomato (Bharti et al., 2000). The up-regulation of *StHsf018* and *StHsf022* was also seen in the leaves of cold-stressed plants as well as *StHsf007, StHsf009*, and *StHsf014*. These data indicated that some of *StHsf* members may have a regulatory role in potato adaptation to heat, drought and cold stresses. These genes showed different expression patterns during different stresses, implying that each *StHsf* may function differently by reacting with different downstream stress protection genes. It is also known that environmental stresses can induce epigenetic and somatic genome variations (Li, 2016) and that Hsfs are among the earliest regulators to stresses, as indicated in the present study. It is interesting to investigate whether Hsfs play a role in directly or indirectly regulating epigenetic and somagenetic variations.

### The co-expression network between potato *Hsfs* and their correlated genes help to excavate the regulatory mechanisms of stress responses

Hsfs have been demonstrated to play key roles in the tolerance to various adverse environment by reacting with different genes especially *Hsps* (Swindell et al., 2007; Hu et al., 2009). The expression of the same *Hsfs* can induce the expression of different groups of *Hsps* and the different Hsfs can transcriptionally activate the expression of the same sets of Hsps under different stresses, which indicated that there exists a complex regulatory network between *Hsfs* and *Hsps*. For example, in *Arabidopsis*, HsfA3 has been confirmed to be in control of the expression of *Hsp70b, Hsp19.9-P*, and *Hsp22.0-ER* under heat treatment while another researches have reported that *Hsp18.1-CI, Hsp26.5–MII*, and *Hsp70* were transcriptionally regulated by HsfA3 in response to drought stress (Sakuma et al., 2006; Yoshida et al., 2008). In this study, Hsf005 (HsfA3) and Hsf007 (HsfA4b) were found to regulate the expression of *Hsp17* members, which suggested that some *Hsps* express under the control of different Hsfs and involve in diverse regulatory pathways (Supplementary Table S3). However, the understanding of precise regulatory mechanisms among Hsfs and Hsps during abiotic stress responses is not clear.

Besides *Hsps*, other genes with different functions are also related to *Hsfs* and induced by various abiotic stresses. Some genes are expressed specifically in response to the distinct stresses and have no significant response to other stresses, while another genes play important roles in the crosstalk of multiple adverse stresses. For example, genes like *DREB1a* and *MAP65* were affected notably by heat stress but did not have striking expression amounts under drought or salt stress (Hu et al., 2009). However, Myb-like DBD protein and CBL-interacting serine/threonine-protein kinase, which were demonstrated to participate in the signal pathways in the tolerance to drought and salt stresses in the previous studies, have also been confirmed to be functional in heat stress (Hu et al., 2009). The potential reason is that various abiotic stresses can trigger the same response mechanisms such as change of membrane permeability (Tsvetkova et al., 2002), production of ROS (reactive oxygen species) (Miller and Mittler, 2006), and consequently generate a lot of protective genes to resist the adverse stresses.

Co-expression analysis, as an important approach to find the new genes and explore the genes potential functions, has already been applied in discovering the genes taking part in the specific biological processes in *Arabidopsis*, maize, and Populus (Higashi and Saito, 2013). The co-expression network between *StHsfs* and their co-expressed genes provided some information and direction to excavate the regulatory mechanisms between *Hsfs* and *Hsf* -associated genes during development and stress responses and can be confirmed by testing the expression changes of detected genes through overexpression or knockout of the corresponding *Hsfs*. Many Hsps like sHsp-CI (PGSC0003DMT400007587), Hsp70 (PGSC0003DMT400008407), Hsp83 (PGSC0003DMT400014217) in module 1 were co-expressed with *StHsf005* (*HsfA3*) (Supplementary Table S3) indicating that Hsf005 plays a significant role in heat resistance (Figure 6A). Another important result that the expression of *StHsf007* (*HsfA4b*) increased markedly under short-time cold stress treatment can be deduced that some genes with the function to respond to cold stress may be induced by Hsf007 (Figure 6C). DELLA proteins, which have been reported to contribute to the increase in freezing tolerance and cold acclimation (Achard et al., 2008; Lee and Thomashow, 2012), can be detected to co-express with *Hsf007* in module 3. This evidence primarily proves our hypothesis and further experiments are necessary to investigate the mechanisms of Hsf007 response to cold stress. Furthermore, in this study, we find MAPK and MAPKKK (Supplementary Table S3) among the co-expressed genes of *StHsf007*. MAPKs (Mitogen-activated protein kinases) are protein Ser/Thr kinases that transform extracellular stimuli to a wide range of cellular responses (Cargnello and Roux, 2012). The MAPK pathway has the highest number of top-ranked proteins among signaling pathways (Du et al., 2012) and is the pathway heavily involved in plant tolerances to stresses (Li et al., 2016). The co-involvement of Hsf007 with the signaling MAPK pathway proteins in the same module suggests that *Hsf007* likely plays a role in the signaling in response to some abiotic stresses. Therefore, *Hsf007* is among the key candidate genes of which the molecular mechanisms in stress tolerance should be further illustrated.

## Conclusions

In summary, a total of 27 *StHsfs* in the *Solanum tuberosum* genome were identified. A series of analyses of these genes, including phylogeny, chromosomal location, gene structure, expression profiling and abiotic stress responses, was performed by bioinformatics and qRT-PCR methods. The *StHsfs* were unevenly distributed in 10 of the 12 chromosomes. The amino acids numbers inserted in HR-A/B domain are based to categorize 27 StHsfs into three large classes. In the phylogenetic tree, the majority of subfamilies contained members from potato, tomato, cucumber, soybean and *Arabidopsis*, which suggested that the structures and functions of most Hsfs were conserved during evolution. A majority of *StHsfs* were found expressed in more than one tissue in potato according to the RNAseq data analysis. The promoter analysis suggested that nearly all StHsfs were activated in response to diverse abiotic stresses. An extensive expression analysis indicated that *StHsf* genes may play various roles in different biological processes in plants: *StHsf004, StHsf005, StHsf009* become predominant *StHsfs* during heat stress; *StHsf004, StHsf007, StHsf009*, and *StHsf014* function as predominant genes under both drought and cold stresses. Furthermore, the co-expression network implied that there is a complex transcriptional regulatory network between *StHsfs* and their correlated genes. Besides, the co-expression network provides some information and direction to excavate the regulatory mechanisms between *Hsfs* and their co-expressed genes during development and stress responses, and also helps to select candidate *Hsf* genes, such as *StHsf005* and *StHsf007*, for future functional analyses.

## Author contributions

RT, QY, and WZ conceived and designed the research. RT, WZ, and XS performed qRT-PCR analysis. RT and XL performed the bioinformatics analysis and construction of co-expression network. RT, XS, XL, JC, and MW analyzed the data. RT and QY wrote the manuscript.

### Conflict of interest statement

The authors declare that the research was conducted in the absence of any commercial or financial relationships that could be construed as a potential conflict of interest. The reviewer SG and handling Editor declared their shared affiliation, and the handling Editor states that the process nevertheless met the standards of a fair and objective review.
