# Supplementary material for: Genome-Wide Identification and Function Analyses of Heat Shock Transcription Factors in Potato
Source: Front Plant Sci. 2016 Apr 19;7:490. doi: 10.3389/fpls.2016.00490 (PMC4836240; doi:10.3389/fpls.2016.00490)
Supplement: Supplementary file 1 [file Table1.DOC]

**Supplementary Table S1. Real-time-PCR primers of *StHsf* genes**

| **Gene Name** | **Forward Primer（5′→ 3′）** | **Reverse Primer（5′→ 3′）** | **Fragment length（bp）** |
| --- | --- | --- | --- |
| StHSF001 | F:CAGAATGGCAATAACAG | R: TTGATACCTGACTACCT | 126 |
| StHSF004 | F:GATCTTCAGCAATCCAACT | R:TCATCGTTAGTCTCCTCTT | 104 |
| StHSF005 | F:GGCTCTCATTTCAAAGG | R:CACCATACTTCCAATAGC | 138 |
| StHSF007 | F:GCGTGTGATATTAGATTAGG | R:GTTGCTGGACTACTAAGAT | 104 |
| StHSF008 | F:ACTTCACTTGGACCTAC | R:CTTCTGACGATGTTCTG | 103 |
| StHSF009 | F:TCAATACTTACGGCTTCC | R:TGACTATGACTGTGAATGG | 127 |
| StHSF012 | F:TCATCAAGGTCCTGTTAG | R:CAAGTGCTTCAAGTTCAG | 116 |
| StHSF014 | F:TAAGGAACACCAGCAGAAT | R:GCATAGCCATAACAATGAAGG | 110 |
| StHSF015 | F:GACAGTGGTTGAGGAAT | R:CATTGTGACTTGCTCTAC | 143 |
| StHSF016 | F:GATTGGACTTCAGAAATGG | R:CTCCTCATCTCTTCCTCTA | 156 |
| StHSF017 | F:CTCCGTAAGTTCCAATG | R:AGTTCCTGCTCAGATTC | 135 |
| StHSF018 | F:TACAACGGTAGAGAATGG | R:GACGATTCATCACAAGAG | 114 |
| StHSF019 | F:GGTTACTGACGATGTGAT | R:GTGCTTGAAGTATTTGGG | 103 |
| StHSF021 | F:GGAACTTATAGAGGAGAAC | R:CGAGTAATTGGACATCAG | 115 |
| StHSF022 | F:GATGGGAGTTCAGTAACGAT | R:GTTGTTTGGTTGTTGCCTAT | 101 |
| StHSF024 | F:TCATATCATACGGGTTCG | R:TGCTCGCATTATTGTTGG | 151 |
| StHSF026 | F:CAACGACGAGGATGAAGT | R:GGACACCACACAACAAATC | 142 |
| StHSF027 | F:TCAACACCTATGGATTCAG | R:TTCTTCGTCTTCACTCTC | 158 |
