# Supplementary material for: Genome-Wide Identification and Function Analyses of Heat Shock Transcription Factors in Potato
Source: Front Plant Sci. 2016 Apr 19;7:490. doi: 10.3389/fpls.2016.00490 (PMC4836240; doi:10.3389/fpls.2016.00490)
Supplement: Supplementary file 4 [file DataSheet1.DOC]

**Supplementary material list**

**Supplementary S1.** The deduced protein sequences of StHsfs. The letters in blue represent the core of a repression sequence; and the red letters in StHsf019 indicate the histone-like motif.

**Supplementary Figure S1.** Multiple sequence alignment of the DNA-binding domains of StHsf proteins. Conserved amino residues are in upper-case letters and are highlighted. DBD contains 3 α helixes and 4 β pleated sheets. The mutated amino acids which might change the structure and function of StHsf015, StHsf016 and StHsf017 in α_3_ region are indicated in red box.

**Supplementary Figure S2.** Multiple sequence alignment of HR-A core and HR-B regions of StHsf proteins. Conversed hydrophobic positions of HR-A core and HR-B are highlighted.

**Supplementary Figure S3.** Expression changes of each StHsf member during heat, drought and cold stress. Values are means ±SD of three biological replicates and are expressed as apparent expression levels relative to a control gene EF1α. Statistical significance in differences between control and stress-treated groups (2 h, 6 h, or 24 h) is indicated by an asterisk. (Note: the expression levels of some StHsfs are too low to be seen in this comparative presentation, but statistical significance in the changes of their transcript levels in response to stress is shown.)

**Supplementary Table S1.** Real-time-PCR primers of *StHsf* genes

**Supplementary Table S2.** The expression of each gene in different organs or tissues extracted from RNAseq data

**Supplementary Table S3.** The Hsf-co-expressed genes sequence number in Spud DB Potato Genomics Resources
