# Supplementary material for: Genome-Wide Identification and Function Analyses of Heat Shock Transcription Factors in Potato
Source: Front Plant Sci. 2016 Apr 19;7:490. doi: 10.3389/fpls.2016.00490 (PMC4836240; doi:10.3389/fpls.2016.00490)
Supplement: Supplementary file 5 [file DataSheet2.DOC]

**Supplementary S1.** The deduced protein sequences of StHsfs.

StHsf001

MDGVHEAPVAGNSSSPPFLNKTYDMVDDPSTDSVVSWSKSNNSFVVLNVPEFARDILPKYFKHNNFSSFVRQLNTYGFRKVDPDRWEFANEGFLRGYKHLLKSISRRKPPQVQGHQQTTSKVPNASVASCVEVGKFGIEEEVERLKRDKDVLMQELVKLRQQQQETDHQLVTVGQRVQLMENRQQQMMSFLAKAMQSPGFVAQLVHQQNGNNRHITGLNKKRRLPEQSEENVACNPVNALQDGQVVRYQPSMTEAAKVLQQQILKINASGRLDNRLNTNDCLTNNPLPPQNSLDSSGASSCISSVTLSEVMSTSSQPNLMSDSGFPFNSCSSIISETQSSTTVVPGEANIPVLPEADALNSQAVHDLPEFSHILGVTDLDLNMSETGNVPDIDTAQGILDGLTTIVSDEFSANTDGDVLLDDMPKLPAINDIFWDQILSATPASPLTGDTDEIGSLVVEEDFLGVQESDWDKLKHMHHLTEQMGLLSSVAQI

StHsf002

MGSTSMDAGGSSPVPEPTPAPMASTNTLPPFLMKTYDMVDDPSTDKIVSWSPTDSSFVIWDPPELAKELLPKYFKHSNFSSFVRQLNTYGFRKVGSDNWKFSNDGFIRGQKHLLKNISRRKPAHGQSQQQQQQQQLHGQSASVGACVEVGNFGIEKEVEWLKRDKNVLMQELVKLRQHQQTTDNQMQTMVQNLQIMEQRQQQMMSFLAKAVNSPGFLAQFVQQQNDNNKRKMEGNKKRRIRQDIPSDDHSVSPADGQLVKYQPIMNEAVKGMLRQIPELDSSPRLENFSNSPESSPTGDAFDGRSNNRISGVTLQEVSHAFSQPFASATSAIAGQSSLSANIQFSESSSLVGAQDLPPILPFSSDMIMPVPSQLQEIVPENNMDINGTERGHDSFMDPTLWGNGKLPLENDIFPPDLQIKWESALIDDIGEPPSVGDPSWEKFLQSLYPTESEEMGSVEIENINTTETESLENGWYNVQHMEHLTEQMGLLTSNTKKV

StHsf003

MSPDGEKTMEGVHEIGNSLPPFLSKTYDMVDDRSTDTVVSWSKSNNSFVVWNVPEFSRDILPKYFKHNNFSSFVRQLNTYGYKKVDPDCWEFANEGFLRGQKHLLKTISRRKPSQMQVHQETASQVQSLSVGSCVEVGKIGIEEEVERLKIDKSIHMEELVNLRQQQKATDHRLENVGQRLQLMEQREQQAMTFLAKALQSPGFIAELVHQQNEGKRRIPGMNKKRRFPNQEEENYAAKQVSTLRDRQIVRYQPLMNEAAKALLQKLLKTNTSGRLETIVKNTHGFLTNRARSFENTLETGGISPHISEVTLSQLATSSQSHLMSDSGFPFNSSLSVIPEIQYSPSLVPGQAKVPQFPELNALNSQTDHVNPEFSGHGFNTPETLEITDLKQPETGDMPYIETMQDIVDDVASIVPDGFSMDDVFLDEMPKLPGINDIFWDQILLASPLTGDKDEIGSLALEDGLAKEEDVPEVQESDCEELKRMSHLT

StHsf004

MEEVVKVKVEEDGIPTAVLPMEGLHDVGPPPFLSKTYEMVEDSSTDEVISWSTTRNSFIVWDSHKFSTTLLPRFFKHSNFSSFIRQLNTYGFRKVDPDRWEFANEGFLGGQKHLLKTIKRRRNVGQSMNQQGSGACIEIGYYGMEEELERLKRDKNVLMTEIVKLRQQQQSTRNQIIAMGEKIERQERKQEQMMSFLAKIFSNPTFLQQYLDKHVQRKDKQRIEVGQKRRLTMTPSAENLQDVATRSDHQPMNYSSHQEREAELASIEMLFSAAMDNESSSNVRSASVVTANGTGPEDIWEELFSEDLISGDRAEEVLVVEQPEFDVEVEDLVAKTPEWGEELQDLVDQLDFL

StHsf005

MNPFDKKQESDTNTKNPFLTEMDSEFAAFSPISLPFADPSSPFINFGSFATPLSQQPVGEGGEIEQLAEEGMGVPHPMECLHGIQIPPFLSKTFDLVEDPLLDSIISWGRNGDSFVVWDPVEFSRLVLPRNFKHSNFSSFVRQLNTYGFRKIDADRWEFANEGFLRGKRHLLKNIQRRKSHQAGSSSGSSAEAGKGTMDEIEKLRNEKSLMMQEVVELQQQQRGTVQQMESVNEKLQAAEQRQKQMVSFLAKVLQNPTFLARVRQMKEQGEITCPRTMRKFVKYQPHGPDGVGSSSMEGQIVKVRSDFQDLAACFESPDFNPVVDQQLPETGLGAEAMPFEGGTVASEELTVAHELFNCSDREIGGASFFNPEGSHFKGKNVASLQLEVMPEYFASFPEEMGKEKNISGFSSPAIGSMVKDEELWSMGFEASAGMPSAGTELWDSLSSYVPDFGVSSGLSDLWDIDPLQAAGSSGVDKWPADGSPFGQSESHANQPKNDSF

StHsf006

MDEAPCSVNALPPFIAKIYEMVDDPSTDPIVSWSSNNKSFIVLNPPDFARDLLPRYFKHNNFSSFIRQLNTYGFKKIDPEQWEFANEDFLRGQPHLLKNIYRRKPVHSHSVQNIHILSSSALTESERQGYKEDIEKLKHENESLHLVLHRHKQDHQGLEMQMQVLNQRVQQVKDRQKNVLSTLARTINKPGLALSLMPQLEMNERKRRLPGNSFLYNETGLEDNQASSSENSTRENMDPTSLLTLNKEVLDQLEPSLTFWEYTLRDIDQARMRQSSSIDLDESISCADSPAISYPQLTVDVGSKVSDIDMNSEPNGNTTPDVTPPENRVETASNNVPTGVNDVFWEQFLTENPGSTDVKPEREDMESKISESKTVEDGKFWWNRKTVISLTEQLGHLTPAERM

StHsf007

MVSIVMDNCNERSSSSSPAPFLLKTYELVDDTYTNPVVSWNHNGRSFVVWNPPEFARDLLPKYFKHNNFSSFIRQLNTYGFRKVDPEQWEFANEEFLRGQRHLLKNIYRRKPIHSHSATGQSVAALTDSERQEYEDEIERLKRENSMLQSSAENQEKFNMEYESGIKSMEQRLQNVAHRQGKLISLLAQLLQRPGFSSDFIQCANNNSKKRRLLVSNYLTEEENAATNSVVAPKLDMEMVKKLDSSINFWERFLYGTRMNPTEDQTCDFDHTQPLPSPIVIREMDTSSDDSGKRNSPIDHSPSSSELGGGPLSPVISSIYVNLECQLKPSDQVNQVEGKTTKTSELVSNSGNDVFWQQFLTETPGCTEPQEVENKGINELACDIRLGDNHRYWWNSGVNVENLAERMGHLSSPATGS

StHsf008

MDEASCSTNALPPFLAKTYEMVDDPSCDAIVSWSSNNKSFIVWNPPDFARDLLPRYFKHNNFSSFIRQLNTYGFRKIDPEKWEFANESNFIRGQPHLLKNIHRRKPVHSHSAQNLHGLSSPLTESERHGYKEDIQKLMHENGSLHLDLQRHKQDHQGLELQMQVLTERVQHAEHRQKTMLSALAQTLDKPVMDLSHMPQLQVNDRKRRFPGNSCLYNESDLEDMRGISSRALSRENMNPSSLLTMNTELLDQLESSLTFWEDVLQDVDQAGIRQNCSLELDESTSCADSPAISYTQLNVDVGPKASGIDMNSEPNANTTPEVAEPEDKAAVAETATNVPTGVNDLFWEQFLTENPGSVDAPEVQSERKDIGSKKNESKPVDSGKYWWNMKSVNSLAEQMGHLTPAEKT

StHsf009

MDVISAAVAAGGGGGPAPFLLKTYEMVDDSQTDDIVSWTPTGHSFVVWNPPEFARILLPTYFKHNNFSSFIRQLNTYGFRKIDPERWEFANEEFLKDQKHLLKNIHRRKPIHSHSHPPGSTVDPERAAFEEEIDKLTREKSGLEANVSRFRQQQSAAKLQLEELTGRVGSIEQRQESLLAFVEKAIQNPDFVERLAQKLESMDISAFSKKRRLPQIDSTQPVQESMSVDNHSSSRVEFGNLSHQDFSNKLRLELSPAVSDINVLSCSTQSSNDDGGSPAHRRISEGWSREVQLRTVGVIYTPEAIELSDTGTSFTLKMDSSLPRASSNVESSRLHPLPQSLTSNEEVDGHISCQLNLSLASCLSQVDKNQYSVRMPQIGQEIGKRFESQSDANDKIPPTDDKSLPPSHDATANKQVPAAAPVRVNDVFWEQFLTERPGCSDNEEASSSYKGNSYDEQDERKSNQGVASNTRKVEHLTL

StHsf010

MDPNFGVPIKEEFPRSNEPSMWIPQPMEGLHENGPPPFLTKTYEFVDDQNTNNVVSWSIGNNSFIVWDPQTFAMNLLPRYFKHSNFSSFVRQLNTYGFRKVNPDQWEFAHEGFLRGQRHLLKTIRRRKTSNFHPGQGSNQGIDSYIELGKLEIDGEIDRLRREKKDLMMELVELKQHQQTTKSNIKSMEEKLKRTEAKQQQMMNFLAKAMQNPRFLEQMMQQKERRKELEEEIKNKRRRQIDHHQGPSNIVGDLDHSVNNSDGNFNIKMEPHEYYYGEMNGFDDLELEASLAMSMQGGPSGNTINFEKGYNIENKSIDHEGFWEGLLNENIEDVINLLEGENEEDHVDILAHQLGFLGSTPK

StHsf011

MDDFDNLIKEEFDGSFLVPQPKECLHENGPPPFLTKTYELVDDPSSNDVVSWSRGNNSFIVWDPQNLAINFLPRYFKHNNFSSFVRQLNTYGFRKVNPEHWEFANGGFLRGEKHLLRTIRRRKTSNFIKSSSSINQGMDSSSSSSSSSSSSSCVELGSFGSFDGEIDELRRDKQVLMIELVKLKQHQKATKSRLQVMEQKLQGTEIKQEKIMSFLAKALQNPNFVEQIMQQKDKRKQLEEAINNKRRRPIDYHEAAACPSKHHNFGNDDHDINNEFDDDLQVAMNMSHGNTLINLEEENNYVEKNNEGFWEDMLDENNVEDEIMALLGVNEQEDEQVHVDVYSD

StHsf012

MMNQLYSVKEEFPGSSSGGGEPPPLTPQPMEGLHDIGPPPFLTKTYEMVDDSTIDHVVSWNRGGQSFVVWDPHAFSTTLLPRFFKHNNFSSFVRQLNTYGFRKIDPERWEFANEAFLKGSKHLLRNIKRRKTPNSPTEQGLGPCVELGRFGFDGEIDRLRRDKQVLMMELVKLRQNQQNTRAYIRSLEVKLQGTERKQQQMMNFLARAMQNPEFVHQLIHQKGKRIEIEEDITKKRRRPIDHQGPVSTLHVGGSSHYIKSEPLEFGEANGFQVSELEALALEMQGFGRARKDQQEEYTIEGLEQFGNTDKELDEGFWEELFNDEDVSGNEDREEEDVDVLAESEFSSFQRRMKKAAIWINQLLVDS

StHsf013

MMNQLYSVKEEFPGSSSGGGEPPPLTPQPMEGLHDIGPPPFLTKTYEMVDDSTIDHVVSWNRGGQSFVVWDPHAFSTTLLPRFFKHNNFSSFVRQLNTYGFRKIDPERWEFANEAFLKGSKHLLRNIKRRKTSNSPTEHGLGPCVELGFDGEVDRLRRDKQVLMMELVKLRQNQQNTRAYIRSLEVKLQGTERKQQQMMNFLARAMQNPEFVQQLIHQKGKRIEIEEDITKKRQRPIDHQGPSASTLHVGGFSHFIKSEPLEFGEANGFQVSELEALALEIQGFGRARKDQQEEYTIEGLEQFVYTDKELDEGFWEELFNDEDVSGNEEEDVDVLAESEFSSIQRQMKKAAIWINQLLVDS

StHsf014

MVKSFENGVSVAPFLLKCYEMVEDESTDGLISWNQSEKSFIIWDVPKFSSELLPKYFKHSNFSSFIRQLNIYGFHKTDTDRWEFLNDSFVKGQKHFLKNIVRRKQSSVAQKKPSQLEEIKSCTSEESKNLELWKEVENLKDERNVLTQELVKLKEHQQNSESKLILLREQLKVREKNQQQMLSFIVMAMQSPSFLVQFFQPKENSWCMSENGNNILSEVEDDCVDTPSDRAIVRYHPPTHEEAAEPPLCAEPEPALDSQKPMELDFSSNELKDMFSNIDFFSGLMDEKLLAFENRVPLTLPYYPDDENLLEQLLLSSPITENKEGEVIDNQACSHAVMETDLSFQPIESGTFSGSEEPFHSLEDKKMEMALLETQSDNLSNMDILTEQLGHSKF

StHsf015

METEGGKGKGKMTEIDDIVEALGESYEGLEDVHIDISMLEDDNDDDDNQCDNRSNEVIHGNAATATPTTPRELFSSSSNRRNFQRRASPFVLKIYEMLADIQFKSLISWSNNGTSFIIHDNHKFAVEVLPRFFRHNNISSFVCQLNSYGFKKVSWDKFEFRHDCFQRGKGQWLRNIKRKISKSQMNEQSTERQAIDETVAFTMEKEIEEIRVEQVTMREEIMMLQRQLDVLEKEMEDINQAGNNMSSKKAKICMILFNSLFACTRGLDSTEVAEEQGGEVEDGVENSGKGNRGKKRKMQVVEELEGDNEGRKKGNPSKKRKMQVVEELEGDNEGRKKIADAADFKTDSYLGRMLMDEMNLNNLAQEQPDNFLESEELAGSSTFWTDYVEKMDHKAISGVDPALSQSQEIPL

StHsf016

MAMNNMNQFSSGEIGHGIGDGAKREPGIAPFVTKTYDMVEDPNSNSIICWSSSGTSFVIWDHNRFSFEVLPKYFKHTNMSSFVYQLNNYGFKKIGLQKWEYGHYWFQAGKKHLLCNIKRRMKNLNAHRDFNQETYFYGVEEEMRSQRDLNITLKSEFEKLKERQDDMVKGIASLKEYLEKSEAESRKFLCFLAKAVKQVVATKRDVEVRDVIMSKRRAEDVMGSSKSSEKKKMIDFSAIKRVHDIM

StHsf017

MEINNQENGGKGGGGRRRGGGAVGRGRPRLTGLRGNSPPPFLVKTYEMVDDPETDPLVHWTSSKTTFLITDPNKFCVEVLPKYFKHSNLSSFIYQLNNYRFRKVCSYKCEYGNPWFRAGKKHWLKNIKSRIQLSKENNPQQGSHSPRVDLVNNNLEEELEKLRNDHISLRVELQKLKDGQENMRSFFPMLLGCGKEKEIRNIMKLLLEKSEVRGDSSSNDTTKRPRLVESPDRVAGSVQDGIGQTSNSAGGSVSSNEKQKEEATAQNAKNREFWEKLFEDDSESKNEGAEESEQELNRSRAMAEIEEMVESKIAMEGEALIAKAAASLNDETEAYLQLWT

StHsf018

MEEETMREEKTMVIDVNENEKNVNGGVVLEVKEEPVIFIDEDDIIGDFTCSLPKPLDGLRDVGPPPFLKKTFEMVDDPNTDSIISWSNNQNSFVVWDPHKFSIHLLPKHFKHNNFSSFIRQLNTYRFRKIDSVRWEFANEGFQKGKKHLLVNIKRRKQYPQQGGGAKSWVGGCCKDGTEAAEIEKLKKDHNTLKMEILKLKQQQESTDTYLATMNERLQNSETKQKYMVIFMAKTFNNPQFVQHLIEKMKQGGKTTTVENGTKKRRLYSDENQEEYTTIKSEIHTLFSCDESSNSPVEEQKGKGNNNSSPEMVSENYILWEKLMEDDMICENGAETDKYQSEIVLELEDLISNPSECKCMP

StHsf019

MSQRTAPAPFLLKTYQLVDDAVTDDVISWNEIGTTFVVWKTAEFAKDLLPKYFKHNNFSSFVRQLNTYGFRKIVPDKWEFANENFKRGQKELLTAIRRRKTVTSTPAGGKSVAAGTSASPDNSGDDIGSSSTSSPDSKNPGSVDTPGKSSQFTDLSDENEKLKKDNQMLSSELVQAKKQCNELVAFLSQYVKVAPDMINRIMSQGTPSGSSLEELVKEVGGVKDLEEQGSYNDNDDKEDDDEKGDTLKLFGVLLKEKKKKRGPDENIDTCGGRGKMMKTTVDYNGPWMKMSSAAGESSKVCN

StHsf020

MTPQPIDRNGGETTAGETQRSVPTPFLTKTYQLIEDQSIDDVISWNEDGSTFIVWNPAEFAKDLLPKYFKHNNFSSFVRQLNTYGFRKVVPDRWEFANDSFRRGERSHLIDIQRRKVVTAIATPSAAATAVAIVAAPPPPPAQPPGTPAPPPPPAQPPVTVSTSDSCEEQVISSNSSAGSTAELLGENERLRLENLQLNKELNSMKKLCGNIYGMMSNYAQPSSSGNQSAESSSPGLKPLDLLRTEQYLGESQVKAVEDGESLEEPEARLFGFSIGMKRVREGEEERTDHAQDLQLQQPGTTDVKSEASDQESNGESEERSWLVQCGGRNQRTCN

StHsf021

MVPSSIEHSGESASGENQRSLPTPFLTKTYQLVDDSAMDELISWNDDGTTFIVWKPAEFARDLLPKYFKHNNFSSFVRQLNTYGFRKIVPDRWEFANDCFRRGEKGLLRDIQRRKISPACTVVSAVVAPAVAVNAAQAVTVAVAPAVRMVSSSNSGDEQVLSSNSSPAATAAAMLRTTTCTTTQELIEENERLRKENAQLNQELNRLRSLCNNVYNLMSNYSVNPADIPARIPEGRALELLDGSGAAFTAAEEDISPRLFGVSIGVKRVKRSNEEESDTVEDQDQVQPHGSDMESEPLDSGSAHHDDQPWIYRLDLG

StHsf022

MEVLEEVQVSDEKSLLEYVVMKKSSPSPFLLKTYMLVEDPATDDVVSWNSDGSAFVVWQPAEFARDLLPTLFKHSNFSSFVRQLNTYGFRKITTSRWEFSNDKFKKGEKNLLREIRRRKAWTNRQQPNNGGQSQDANSNNKKDNITEEDQRSSSSTSSSSEYINLVDENKRLKMENGVLSSELSLMKNKCKELMNIVTIFAKIPEKEEEKKPMLFGVRLEVKEEMERKRKRVELNEIASVFLSQLCK

StHsf023

MGELEDECERNLLEYVRKASTSPFLLKTYMLVEDPATDDVISWNSDGSAFIVRQPAEFARDLLPTLFKHSNFSSFVRQLNTYGFRKVTTSQWEFSNDLFRKGEKDLLCDIRRKKAWTNKQQPPNKNNKKESEDEDQKSSSSTSNSSSSFEYNSLVDENKRLKMENGELSYELSLVNKKCKELIDLVAILLKNSNEEEKKEGQKGKRPMLFGVRLEVEEEMERKRKKVELNEIASVFLSQTMQIK

StHsf024

MALMLDNCEGILLSLDSHKSVPAPFLTKTYQLVDDPSTDHIVSWGEDDSTFVVWRPPEFARDLLPNYFKHNNFSSFVRQLNTYGFRKIVPDRWEFANEFFKRGEKHLLCEIHRRKTAQPIQNMSMNPHHSYHTGSGFFPNYPTNYNPRHSISPPDSDEQFFQQQNINWCDSPSSNNNASNNNFTNINTNTNTVTALSEDNDRLRRSNNMLMSELAHMRKLYNDIIYFVQNHVKPVTPSSSYNTCSLLPASATPIVQKNMNMNIHHQFGYQQITNPKNVAISNINNNNNNNNVVSPSKTSQSSSVTILDEGNGGGDNRSTKLFGVPLMSKKRVHPEYSSSYYSTTNMVETNKARLMVLEKNDLGLNLMPPSSS

StHsf025

MASSTCCDQEMFLTMQKAMVAPFLTKTYQLVDDPSSDHIVSWGEYETTFVVWRPPEFAKDLLPNYFKHNNFSSFVRQLNTYGFKKIVPERWEFANEYFKKGQKHLLCEIQRRKSTPQQLHNLTPYHEHITRSIYHQETQNPSSVDHDILLALTQDNERLRKRNIVLLSELTHMKNLYNDIIYFIQNHIKPVEEKFSVFLLNEDLNQKIVDHHRHRQVHIEDHEEPKSDKVIPSTRYLHVVVDGPKRLKNFEDASVIPVEPLLCESTWDIPSYVSEPKLMSPGSKRTVGRPQLKRWKGFADVKFKRFSDRSRRRFFIVRFVKRLILIDCVSTCFKGLIKITEIGCRISEGEASNKSALNTASPNEVWKHFEKRKM

StHsf026

MATDNRNIHRKIGRSTTRMKCPAPFLSKTYDLLEEQKEENNNNNIKVVSWNGEGNGFVVWCPDEFSEVMLPKYFKHNNFSSFIRQLNTYGFKKVASKRWEFHHEKFQKGCRHLLAEITRKKCEPSVFPTYLNPSKKSTLLLARNNQENTTRQLLMEENQNLKKEKMELQLQIAHFKTLEMKLLQCLSQCVENPHNKTRRLF

StHsf027

MEANNIIAPFVMKTYQMVNDPKIDGLIAWGTANNSFIVVEPLDFSQRILPVYFKHNNFSSFVRQLNTYGFRKVDPDKWEFANEWFLRGQTHLLKNIVRKKHSRNSCSQKQDESEDEEIFTEISRLKQEQKVLDHEVENMTRRLEATEKRPQQMMAFLCKVVDDPEILPRILMEKEKSKRLSLTNREKKRRLMISNSTSCSSVKSEDAVGATSSFHSPDANFDKDAICQSSPSSGTPPPPAWLGCRPMITYEPGTIQSIRSPDANFDKDTFYQSSPSSETPSTAWMSQQKVVIGGRPMMMTNEAYGSCPTMSSTLSTGSSESGGFTAPPMDNFYGYDYGGGGGGGGGGTTATEEASPPPYPFSLFGGGF

**The letters in blue represent the core of a repression sequence; and the red letters in StHsf019 indicate the histone-like motif.**
