# Supplementary figures and images for: Genome-Wide Identification and Function Analyses of Heat Shock Transcription Factors in Potato
Source: Front Plant Sci. 2016 Apr 19;7:490. doi: 10.3389/fpls.2016.00490 (PMC4836240; doi:10.3389/fpls.2016.00490)

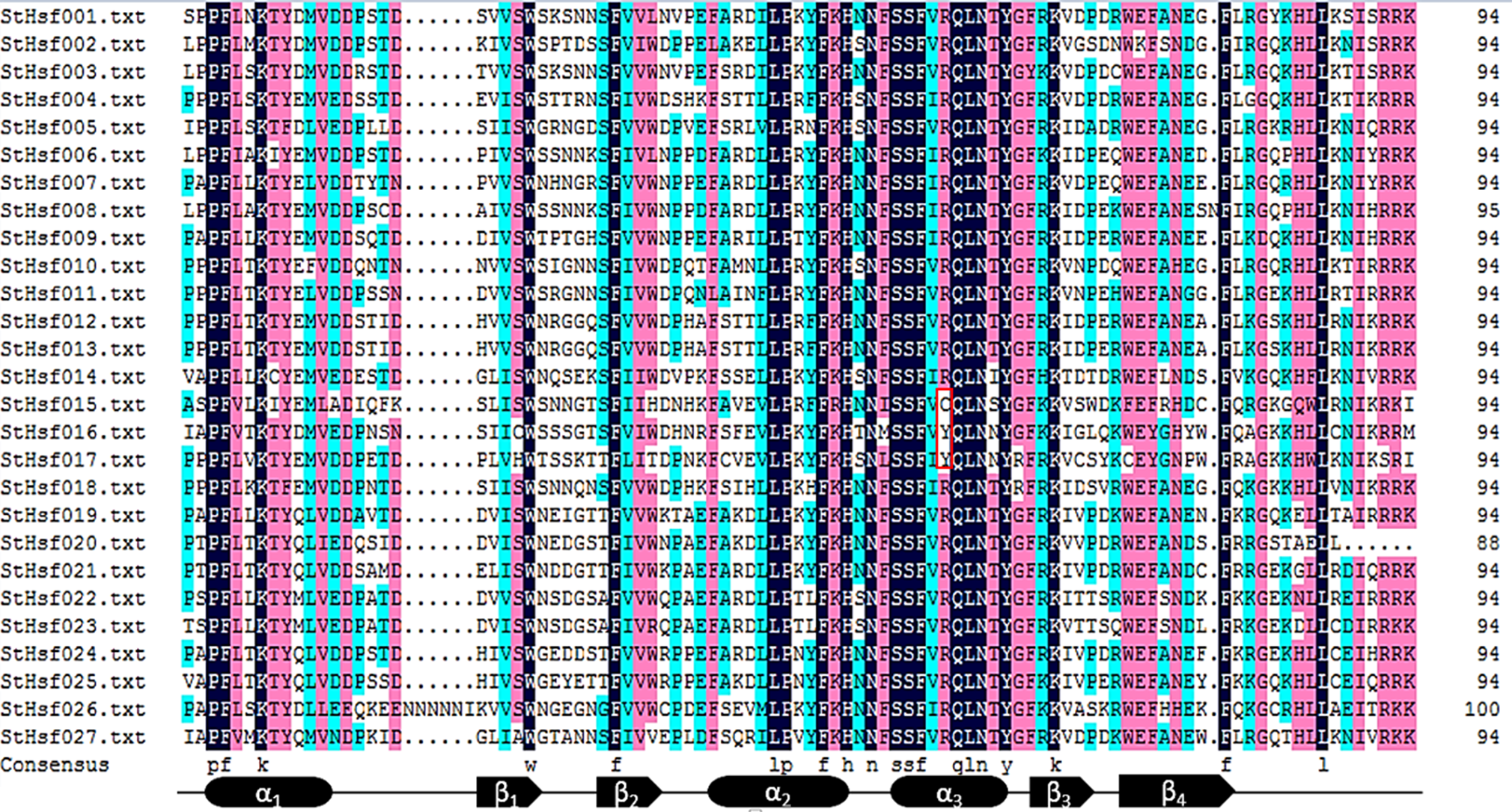

Supplement: Supplementary file 6 [file Image1.TIF]

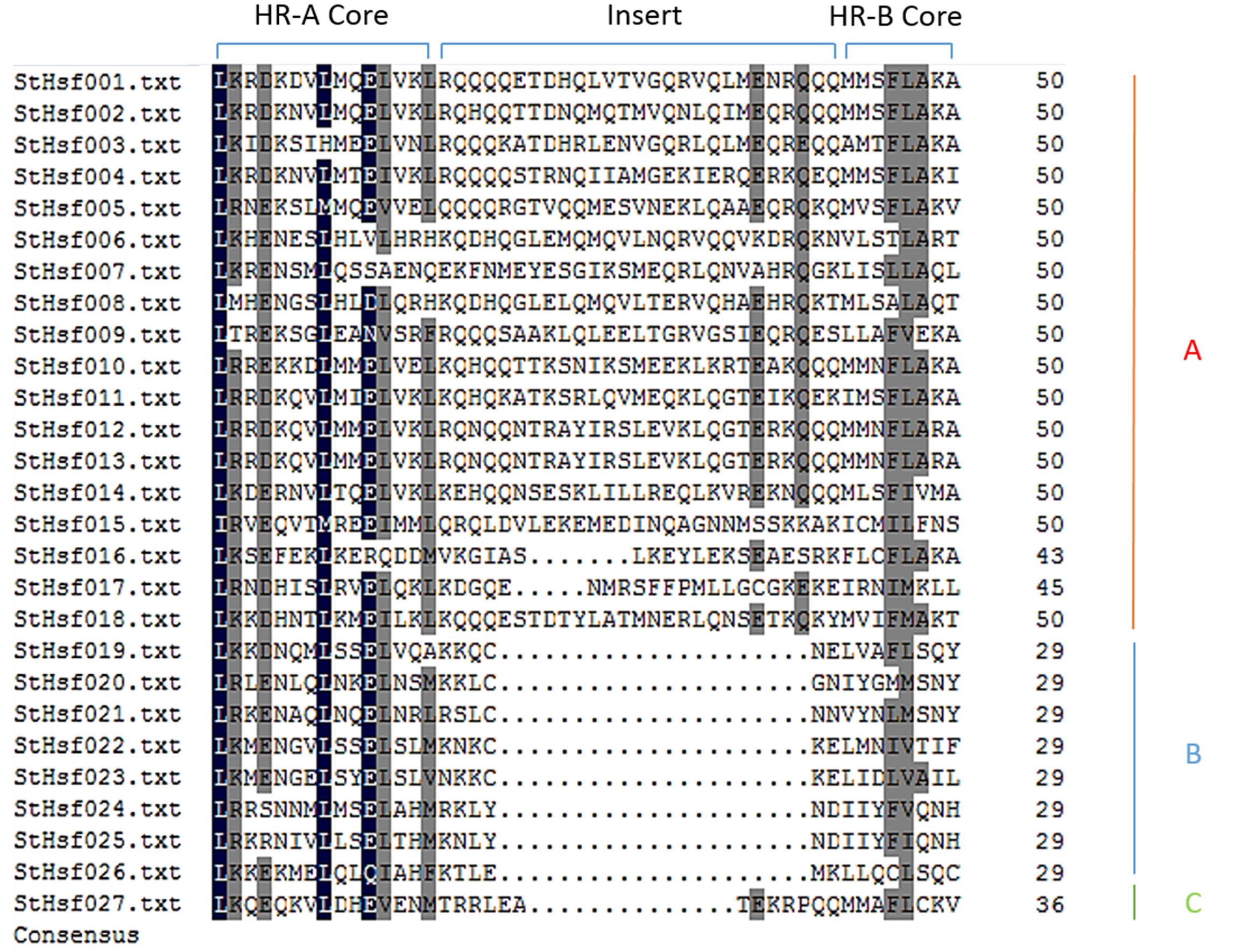

Supplement: Supplementary file 7 [file Image2.TIF]

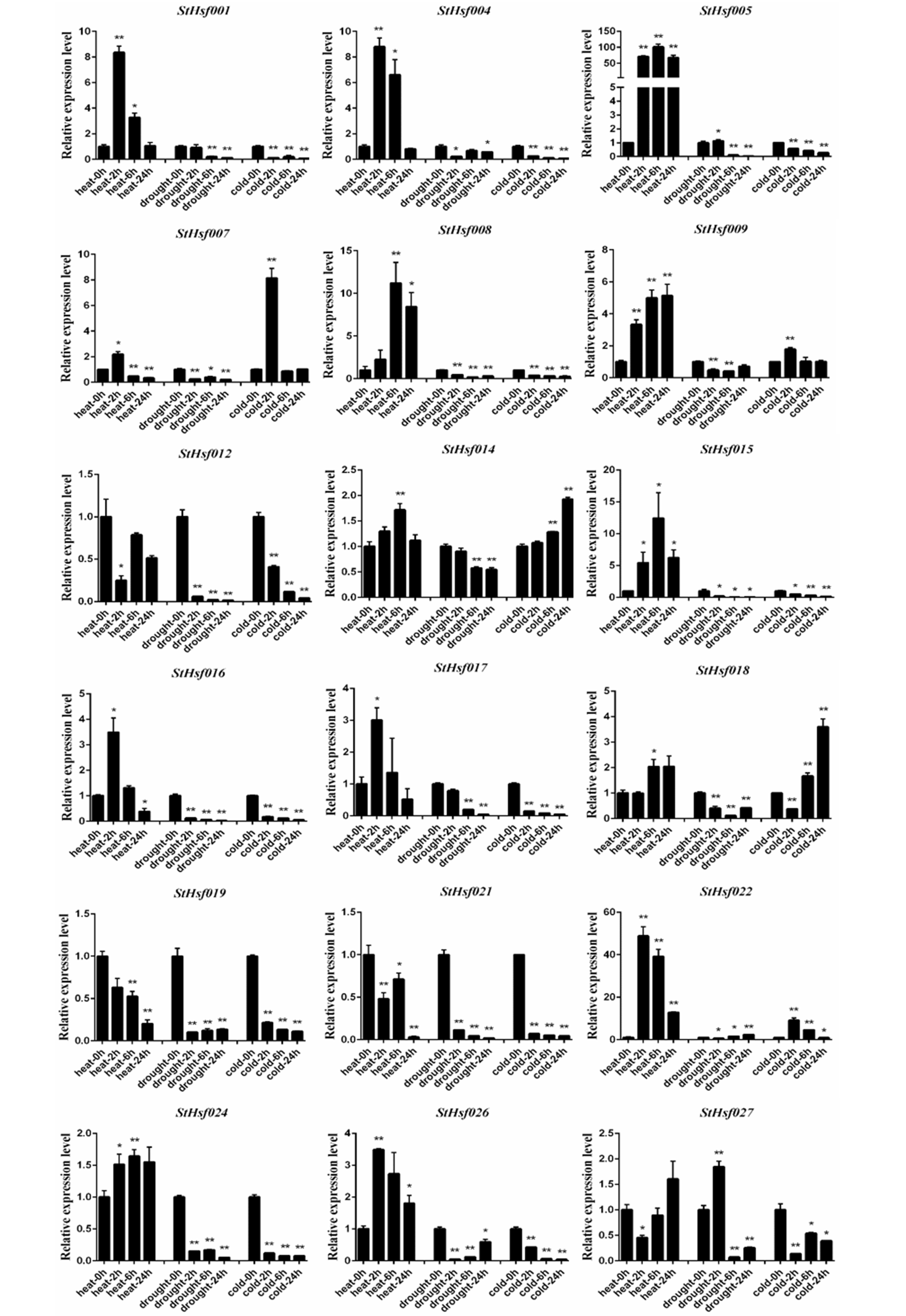

Supplement: Supplementary file 8 [file Image3.TIF]
